# An improved method for high-throughput quantification of autophagy in mammalian cells

**DOI:** 10.1038/s41598-020-68607-w

**Published:** 2020-07-22

**Authors:** Lennart Koepke, Benjamin Winter, Alexander Grenzner, Kerstin Regensburger, Susanne Engelhart, Johannes A. van der Merwe, Stefan Krebs, Helmut Blum, Frank Kirchhoff, Konstantin M. J. Sparrer

**Affiliations:** 1 Institute of Molecular Virology, Ulm University Medical Center, 89081 Ulm, Germany; 2 Gene Center and Laboratory for Functional Genome Analysis, Ludwig-Maximilians-University, 81377 Munich, Germany

**Keywords:** Autophagy, Virology, High-throughput screening, Immunological techniques, Microbiology techniques, Microscopy, Sensors and probes

## Abstract

Autophagy is a cellular homeostatic pathway with functions ranging from cytoplasmic protein turnover to immune defense. Therapeutic modulation of autophagy has been demonstrated to positively impact the outcome of autophagy-dysregulated diseases such as cancer or microbial infections. However, currently available agents lack specificity, and new candidates for drug development or potential cellular targets need to be identified. Here, we present an improved method to robustly detect changes in autophagy in a high-throughput manner on a single cell level, allowing effective screening. This method quantifies eGFP-LC3B positive vesicles to accurately monitor autophagy. We have significantly streamlined the protocol and optimized it for rapid quantification of large numbers of cells in little time, while retaining accuracy and sensitivity. Z scores up to 0.91 without a loss of sensitivity demonstrate the robustness and aptness of this approach. Three exemplary applications outline the value of our protocols and cell lines: (I) Examining autophagy modulating compounds on four different cell types. (II) Monitoring of autophagy upon infection with e.g. measles or influenza A virus. (III) CRISPR/Cas9 screening for autophagy modulating factors in T cells. In summary, we offer ready-to-use protocols to generate sensitive autophagy reporter cells and quantify autophagy in high-throughput assays.

## Introduction

Macroautophagy (hereafter called autophagy) is a catabolic cellular process^1–3^. Basal levels of autophagy are required to maintain homeostasis of eukaryotic cells, mediating continuous turnover of organelles and proteins. Autophagy is active in almost all cells of the body. Upon induction of autophagy, cytoplasmic cargo is engulfed by double-layered membranes, which upon elongation and closure form so-called autophagosomes, eventually fusing with lysosomes. Their contents along with the inner membrane are degraded at a low pH by lysosomal peptidases and hydrolases. This process is either specifically (selective autophagy) targeting compounds recognized by so-called autophagy receptors like SQSTM1 (p62) or leads to bulk degradation of parts of the cytoplasm^4^. Specific autophagy targets are usually earmarked for destruction by ubiquitin chains. Overall, autophagy primarily has a pro-survival, cytoprotective role^5^. Diverse cellular signaling pathways are regulated by autophagy, including the turnover of key signaling components^6^. Furthermore, cellular proteins processed by autophagy may lead e.g. to the release of anti-microbial peptides^7^. In addition, autophagy targets foreign compounds such as viral proteins in the cytoplasm (Xenophagy). Thus, it acts as an innate immune defense pathway^8–10^ and eventually provides peptides for display on major histocompatibility complex (MHC) molecules on antigen-presenting cells^11^.

Recent studies revealed that aberrant autophagy impacts several disorders^12,13^. Diseases like cancer and neuropathies are characterized by dysregulation of autophagy^14,15^. Hereditary diseases like Crohn’s disease are linked to mutations in e.g. autophagy-related protein 16L (ATG16L), a protein necessary for autophagy induction^16^. Moreover, autophagy is involved in the immune defense system targeting intracellular viruses, parasites, or bacteria and impaired autophagy may be detrimental during microbial infections^8–10,12^. Therefore, there is a growing need to understand the molecular basis of autophagy in human diseases and upon microbial challenge. Importantly, exogenous modulation of autophagy could be key to novel therapies. Indeed, cancer therapy is already successfully aided by drugs that, depending on the type and stage of cancer, induce or block autophagic flux, such as Rapamycin and Chloroquine^17,18^. However, the currently available drugs lack specificity. Therefore, novel compounds that modulate autophagy in a more specific manner need to be identified^19^. To discover novel regulators of autophagy (both cellular and exogenous), robust, rapid, and cost-effective experimental approaches are necessary. Unfortunately, activation of autophagy does not result in transcriptional activation, so classical reporter-gene approaches are not applicable.

One major feature of active autophagy is the presence of processed ubiquitin-like ATG8-family members that decorate the membrane of autophagosomes^20^. ATG8-family members are modified in two steps. First, proteolytic cleavage occurs, driven by ATG4, that exposes the C-terminal glycine. In the second step, phosphatidylethanolamine (PE) is covalently attached to the final glycine of ATG8 proteins in a ubiquitin-like conjugation catalyzed by the ATG5-12/16L complex^21,22^. The human genome encodes several ATG8 homologs (GABARAP, GABARAPL1, GABARAPL2, MAP1LC3A, MAP1LC3B, MAP1LC3B2, and MAP1LC3C), with Microtubule-associated proteins 1A/1B light chain 3B (MAP1LC3B or short LC3B) being the most prominent member^23,24^. The conversion of LC3B-I to LC3B-II has long been recognized as a reliable marker for autophagosome quantification^24^. For example, conversion of LC3B can be monitored by western blotting as LC3B-I and II migrate at different sizes, with the more hydrophobic LC3B-II traversing faster through a gel. Furthermore, the recruitment of LC3B to autophagosomes can be observed using microscopy as puncta formation^24^. However, a lot of classical methods to study autophagy suffer from human bias, especially the manual counting of enhanced green fluorescence protein (eGFP)-LC3B puncta, high variability, and labor-intensive experimental procedures and are thus not apt for high-throughput applications^24,25^. Recent years have developed robust systems to study autophagy, that have already been successfully adapted for screening approaches, identifying novel parts of the autophagic machinery, regulation as well as drugs that modulate autophagy^26–32^. Eng et al. (2010) reported an easy and rapid method to quantify autophagosomes using flow cytometry, based on the quantification of lipidated, autophagosome-bound LC3B-II. To isolate the membrane-bound eGFP-LC3B-II, soluble, cytoplasmic eGFP-LC3B-I is removed from cells, using mild permeabilization and subsequent washing that retains the membrane(= autophagosome) associated eGFP-LC3B-II inside the cells^33^.

We adopted this system for high-throughput experiments and present here a detailed protocol for robust, rapid, sensitive, and cost-effective measurement of autophagy using a simple flow-cytometry based readout. In addition, we provide details on the construction, selection, and cultivation of clonal autophagy reporter cell lines to assess cell-type specific differences in autophagy. In our system, the reporter construct is stably integrated into the genome of the target cell and no additional selection to maintain stable eGFP-LC3B expression is required. We generated and thoroughly characterized eGFP-LC3B expressing autophagy reporter cell lines based on HEK293T, HeLa, Jurkat, and THP-1 cells. These reporter cell lines allow quantification of autophagy with high sensitivity and are suitable for high-throughput approaches. We further provide examples and experimental conditions on how to use these eGFP-LC3B expressing cell lines for rapid flow cytometry-based quantification of autophagy to discover novel drug candidates, analyze time-dependent modulation of autophagy by viral infections, and identify key factors in autophagy by overexpression or knock out CRISPR/Cas9 analysis.

## Materials and methods

### Cell culture and viruses

All cell lines (HEK293T, HeLa, Jurkat, THP-1) were purchased from American type culture collection (ATCC: #CRL-3216, #CCL-2, #TIB-152, #TIB-202). HEK293T and HeLa cells were cultivated in Dulbecco’s Modified Eagle Medium (DMEM, Gibco) supplemented with 10% (v/v) fetal bovine serum (FBS, Gibco), 100 U/ml penicillin (PAN-Biotech), 100 µg/ml streptomycin (PAN-Biotech) and 2 mM L-glutamine (PAN-Biotech) (hereafter called DMEM + 3). Jurkat and THP-1 cells were cultivated in Roswell Park Memorial Institute (RPMI, Gibco) 1640 medium supplemented with 10% (v/v) FBS (Gibco), 100 U/ml penicillin (PAN-Biotech), 100 µg/ml streptomycin (PAN-Biotech) and 2 mM L-glutamine (PAN-Biotech). Cells were tested for mycoplasma contamination by polymerase chain reaction (PCR) test and used if negative. Measles virus (MeV, Schwarz strain) was a generous gift from K.-K. Conzelmann (Max von Pettenkofer Institute, Ludwig-Maximilians-University Munich, Germany). Influenza A virus (strain PR8/34 H1N1) and encephalomyocarditis virus (EMCV, EMC strain) were purchased from ATCC (#VR-95, #VR-129B).

### Plasmids and transfections

pEGFP-N1_hTRIM32 was a gift from Martin Dorf (Addgene, #69541)^34^. The open reading frame (ORF) of TRIM32 was subcloned into the pIRES_FLAG vector using Gibson assembly (New England Biolabs). The insert was amplified by PCR (Phusion High-Fidelity DNA Polymerase, Thermo Scientific) with primers 1 and 2 (1: GCT CTT AAG GCA GCT AGC GCC CTC GAG GCC ACC ATG GCT GCA GCA GC, 2: CTT GTA GTC TCT AGA TGC ACG CGT TGG GGT GGA ATA TCT TC, Biomers.net GmbH), and the vector linearized with NotI and MluI restriction enzymes (New England Biolabs). pBabe-mCherry-eGFP-LC3B and pCSC-SP-PW-GFP (aka pBOB-eGFP) were kindly provided by Jayanta Debnath (Addgene, #22418) and Inder Verma (Addgene, #12337), respectively. pMDLg, RSV-Rev, and pMD.G together with the generated pBOB construct were used to rescue 3^rd^ generation lentiviruses as previously described^35^. pSicoR-CRISPR-PuroR was kindly provided by Robert-Jan Lebbink^36^. ATG5 and non-targeting single guide RNA coding sequences were inserted by cutting the backbone with BsmBI and subsequent Gibson assembly (NEB) with oligonucleotides (NT: GTG GAA AGG ACG AAA CAC CG ACG GAG GCT AAG CGT CGC AAG TTT TAG AGC TAG AAA TAG, ATG5-1: GTG GAA AGG ACG AAA CAC CGA ACT TGT TTC ACG CTA TAT CGT TTT AGA GCT AGA AAT AG, ATG5-2: GTG GAA AGG ACG AAA CAC CGT TCC ATG AGT TTC CGA TTG AGT TTT AGA GCT AGA AAT AG) to create pSicoR-CRISPR-PuroR-NT, pSicoR-CRISPR-PuroR-ATG5-1, and pSicoR-CRISPR-PuroR-ATG5-2. For all transfections, Polyethylenimine (PEI, 1 mg/ml in H_2_O, Sigma-Aldrich) or Lipofectamine RNAiMax (Invitrogen) was used as detailed in the protocol.

### siRNA mediated knockdown

HeLa eGFP-LC3B reporter cells were grown in 24-well plates and 18 h post-seeding, the cells were transfected with siRNA (Horizon Discovery) either targeting autophagy-related genes (ATG) or non-targeting as a negative control (D-001206-14-20, M-005049-00-0,005, M-015375-01-0,005, M-005789-01-0,005, M-004374-04-0,005, M-020112-01-0,005, M-014294-02-0,005, M-010212-02-0,005). The transfection was done using Lipofectamine RNAiMAX (Invitrogen) following the manufacturer’s instructions. 72 h post transfection, Chloroquine (1 µM, 4 h) was added to one set of wells, transfected with non-targeting control siRNA. Subsequently, the cells were harvested, treated with saponin, fixed using PFA, and analyzed in flow cytometry.

### CRISPR/Cas9-mediated knockout

Third generation lentiviral particles^35^ were generated as described in the detailed protocol 1.2. The pBOB-eGFP-LC3B plasmid was exchanged for the respective pSicoR-CRISPR-PuroR CRISPR/Cas9^36^ constructs harboring ATG5 targeting sgRNAs or non-targeting (NT) sgRNAs (NT: ACGGAGGCTAAGCGTCGCAA, ATG5-1: AACTTGTTTCACGCTATATC, ATG5-2: TTCCATGAGTTTCCGATTGA)^37^. Three days post-transduction, the medium of the transduced HeLa eGFP-LC3B cells was changed to DMEM + 3 containing 0.75 µg/ml puromycin for selection. The selected cells were grown to confluency and differences in autophagic flux between the pools were analyzed by flow cytometry.

### Whole-cell lysates

Whole-cell lysates were prepared by collecting cells in Phosphate-Buffered Saline (PBS, Gibco). The cell pellet (500 g, 4 °C, 5 min) was lysed in transmembrane lysis buffer (150 mM NaCl, 50 mM 4-(2-hydroxyethyl)-1-piperazineethanesulfonic acid (HEPES) pH 7.4, 1% Triton X-100, 5 mM ethylenediaminetetraacetic acid (EDTA)^10^) by vortexing at maximum speed for 30 s. Cell debris was pelleted by centrifugation (20,000 g, 4 °C, 20 min), and the cleared supernatants (SN) stored for analysis at -20 °C.

### SDS PAGE and immunoblotting

Whole-cell lysates were mixed with 6xSDS-PAGE loading buffer (187.5 mM Tris–HCl pH 6.8, 75% (v/v) Glycerol, 6% (w/v) SDS, 0.3% (w/v) Orange G, 15% (v/v) 2-mercaptoethanol) and heated to 95 °C for 5 min. Samples were loaded on a precast 10% NuPAGE Bis–Tris gel (Invitrogen) and run for 2 h at 80 V in 1 × MES SDS running buffer (Invitrogen). Subsequently, the gel was blotted onto methanol activated PVDF-Membrane (Immobilon-FL, Merck) using 1xSemi dry blot transfer buffer (Alfa Aesar) for 30 min at 30 V. Afterwards, the membrane was blocked using 0.5% (w/v) casein (Thermo Scientific) for at least 1 h at room temperature (RT), then incubated with primary antibodies, diluted in 0.05% (w/v) casein (Thermo Scientific) for 1 h at RT or overnight at 4 °C. The membrane was extensively washed with PBS-T (PBS containing 0.2% (v/v) TWEEN-20, Sigma-Aldrich). The respective secondary antibodies (IRDye Secondary Antibodies, Li-Cor), diluted 1:20,000 in 0.05% (w/v) casein (Thermo Scientific), were incubated with the membrane for 30 min at RT, followed again by extensive washing with PBS-T for 30 min. The blot was developed on an Odyssey 9120 (Li-Cor) infrared imager. The following antibodies were used: anti-LC3B (1:1,000, #2775, Cell Signaling Technology), anti-GFP (1:10,000, ab290, Abcam), anti-p62 (1:1,000, NBP1-48320SS, Novus Biologicals) and anti-β-actin (1:10,000, AC-15, Invitrogen).

### Confocal microscopy

HeLa or HEK293T eGFP-LC3B reporter cells were grown on coverslips (VWR) in 24-well plates and treated as indicated. THP-1 or Jurkat eGFP-LC3B reporter cells were grown in 24-well plates and treated as indicated. Cells were fixed with 4% (w/v) paraformaldehyde (PFA, Santa Cruz) for 20 min at RT, permeabilized with 0.5% (v/v) Triton-X-100 (Sigma-Aldrich) in PBS and then blocked with 5% (v/v) fetal bovine serum (Gibco) in PBS for 1 h at RT. Adherent cells were mounted on microscope slides (VWR) in 4′,6-diamidino-2-phenylindole (DAPI, VWR)-containing Mowiol mounting medium (Mowiol 4–88 10% (w/v, Carl Roth), glycerol 25% (w/v, Sigma-Aldrich), H_2_O 25% (v/v), 0.2 M Tris HCl pH 8.5 50% (v/v, AppliChem GmbH, VWR), DABCO 2.5% (w/v, Carl Roth)^10,38^) to co-stain nuclei. The suspension cells were pelleted (300 g, 3 min), suspended in the same mounting medium, and mounted between coverslips and microscope slides (VWR). All laser scanning images were acquired on a Zeiss LSM 710 confocal microscope. The total area of cytoplasmic eGFP-LC3B puncta in HeLa, HEK293T, Jurkat and THP-1 cells was determined using a custom ImageJ macro for ≥ 30 randomly selected cells.

### Flow cytometry and cell sorting

Please see the detailed protocol for the exact experimental procedures. Single cells to create clonal cell lines were sorted into 96-wells using BD FACSAria III. To measure eGFP or allophycocyanin (APC) fluorescence, a BD FACSCanto II or Beckman-Coulter CytoFLEX with attached high-throughput samplers were used. Voltage/gain for forward and sideward scatter were set to such values that allowed clear separation of the cell population from debris. For all co-staining, we chose APC as the fluorophore to avoid compensation with the eGFP signal. eGFP mean fluorescence intensity (MFI) in mock cells was set to above 1,000 to allow for linear monitoring of changes in both directions (less/more autophagosomes) using voltage/gain settings. A minimum of 10,000 intact single cells were measured per sample. Raw fluorescence-activated cell sorting (FACS) data were analyzed using FlowJo 10. Intact cells were gated in FSC-A vs SSC-A and single cells gated in FSC-H vs FSC-A. Stringent gating strategies to exclude debris or dead cells that exhibit autofluorescence were applied. eGFP-MFI of all samples was calculated and eGFP-MFI of the mock control subtracted (= baseline autophagic flux) from the other samples to depict the eGFP-MFI shift (= shift of autophagic flux).

### DNA analysis

sgRNA PCR amplicons were mixed with 6 × loading dye (Roti-Load, Carl Roth) and loaded on a 1% agarose gel (w/v, Sigma Aldrich) in 1 × TAE buffer (Carl Roth) next to a 1 kb Plus DNA ladder (Thermo Scientific). The samples were run for 25 min at 140 V, stained using ethidium bromide (AppliChem GmbH), and visualized in a Bio-Rad Gel Doc XR + .

### Next-generation sequencing and analysis

Sequencing was performed on the Illumina Genome Analyzer in the GeneCenter sequencing facility (LAFUGA). Obtained sequences were processed with the Trim Galore! toolkit to remove adapter sequences and reads with PHRED scores below 30 as previously described^39,40^. Default settings were used to process the raw reads. Combined from three independent experiments, 6.4 million reads were obtained for the low sample and 4.2 million for the input sample. To align the reads to the GeCKO 2.0 gRNA table, quantify, and normalize them, the MAGeCK suite was used^41^. The raw sequencing data were deposited in GEO (ID: GSE147488).

### Statistical and bioinformatic analysis

Data were collected at least in triplicates for all flow cytometry-based approaches and with at least 30 replicates for fluorescence confocal microscopy-based approaches. For the relative comparison of changes in autophagic flux, statistical differences were assessed by one-way ANOVA or unpaired t-test as indicated. Results were graphed by Graph Pad Prism 8, and are displayed as means ± standard error of the mean (SEM) with individual values shown. *P*-values ≤ 0.05 were considered statistically significant. A p-value below 0.05 is annotated with *, p-values between 0.05 and 0.01 with **, and p-values below 0.001 with ***. To evaluate the quality of the method for high-throughput screening, we calculated the Z-factor as described previously^42^. A Z-factor above 0 indicates a method suitable for high-throughput approaches, 1 represents the maximum quality reachable.

## Detailed protocol

### Generating autophagy reporter cells

A detailed list of materials is provided in Table 1.

**Table 1:** Materials to generate autophagy reporter cell lines.

Product	Source	Product number
Phusion high-fidelity DNA polymerase (2 U/µL)	Thermo Scientific	F530
dNTP Mix (10 mM each)	Thermo Scientific	R0191
Dimethyl sulfoxide	Merck	102952
Restriction Enzymes	New England Biolabs	
Silica Bead DNA Gel Extraction Kit	Thermo Scientific	K0513
DNA Ligation Kit Ver.2.1	Takara	6022
Polyethylenimine, branched	Sigma-Aldrich	408727
Opti-MEM I Reduced Serum Medium	Gibco	31985047
Millex-HA Syringe Filter Unit, 0.45 µm, mixed cellulose esters, 33 mm, ethylene oxide sterilized	Merck	SLHA033SS
Trypsin 0.05%/EDTA 0.02% in PBS, w/o: Ca and Mg	PAN-Biotech	P10-023100
Falcon Round-Bottom Tubes with Cell Strainer Cap, 5 mL	Falcon	38030
HEK293T cells	ATCC	CRL-3216
HeLa cells	ATCC	CCL-2
Jurkat cells	ATCC	TIB-152
THP-1 cells	ATCC	TIB-202
**Media**		
RPMI 1640 Medium	Gibco	21875034
Gibco DMEM, high glucose, no glutamine	Gibco	11500416
Fetal Bovine Serum, qualified, Brazil	Gibco	10270106
Penicillin–Streptomycin, 10,000 U/ml Penicillin, 10 mg/ml Streptomycin	PAN-Biotech	P06-07050
L-Glutamine 200 mM	PAN-Biotech	P04-80050
**Verification**		
Anti-GFP antibody	Abcam	ab290
Anti-LC3B antibody	Cell Signaling Technology	#2775
Anti-β-actin antibody	Invitrogen	AM4302
Paraformaldehyde solution 4% in PBS	Santa Cruz Biotechnology	sc-281692
Chloroquine diphosphate salt	Santa Cruz Biotechnology	sc-205629
InSolution Rapamycin	Merck	553211

### General considerations

For the construction of the cell lines use low passage numbers. For accurate autophagy measurements, make sure that the cells never overgrew, even during the construction of the cell lines, discard any flasks which are too dense. Cell lines should be discarded once the passage number is higher than 20 (assuming a biweekly split-cycle).

1.1 Amplify a fused eGFP-LC3B ORF from the pBabe-mCherry-eGFP-LC3B template and insert it into the pBob-eGFP vector, replacing the existing eGFP ORF:
  1.1.1. Set up a PCR reaction to amplify the eGFP-LC3B fragment using Primers 3 & 4 (3: GTG GGA TCC GCC ACC ATG GTG AGC AAG GGC GAG GAG CTG TTC ACC, 4: CGC GTT TAA ACT TAC ACT GAC AAT TTC AT) and pBabe-mCherry-eGFP-LC3B as a template according to Tables 2 and 3.

**Table 2:** Phusion HF (Thermo Scientific) pipetting scheme.

	Volume per tube
5 × Phusion HF Buffer	10 µl
10 mM dNTPs	1 µl
fwd Primer (10 pmol/µl)	1 µl
rev Primer (10 pmol/µl)	1 µl
Template DNA (0.1 µg/µl)	1 µl
Dimethyl sulfoxide	1.5 µl
Phusion HF DNA Polymerase	0.5 µl
ad 50 µl ddH_2_O

**Table 3:** Phusion HF PCR conditions.

	Temperature	Duration
Initial denaturation	98 °C	30 s
35 amplification cycles	98 °C	5–10 s
	55 °C (adjust according to primer properties)	10–30 s
	72 °C	15–30 s/kb
Final extension	72 °C	10 min
Hold for storage	4 °C	∞
  1.1.2. Digest the amplified insert (ca. 1,100 bp) with BamHI and PmeI (both New England Biolabs), purify it on an agarose gel, and recover the DNA (Silica Bead DNA Gel Extraction Kit, Thermo Scientific) to create the insert for ligation.
  1.1.3 Digest the pBob-eGFP vector using BamHI and PmeI, purify on an agarose gel and recover the DNA (Silica Bead DNA Gel Extraction Kit, Thermo Scientific) to create the backbone for ligation.
  1.1.4. Ligate the insert and backbone (DNA Ligation Kit, TaKaRa) to create the pBob-eGFP-LC3B vector.
    **Optional**: An internal ribosomal entry site (IRES) element can be inserted after the eGFP-LC3B fusion ORF to allow the expression of an antibiotic resistance protein. To avoid selection using antibiotics and stressing the autophagy reporter cells, we decided to omit this.
1.2. Generate third generation lentiviral particles (VSV-G pseudotyped)^35^ for transduction of target cells: Transfect HEK293T cells in 6-well plates (about 70% confluency) using PEI (1 mg/ml in H_2_O, Sigma-Aldrich).
  1.2.1. Mix A: Opti-MEM (300 µl per well of the 6-well plate, Gibco) and PEI (2 µl PEI per µg DNA).
  1.2.2. Incubate Mix A at RT for 5 min.
  1.2.3. Mix B: Opti-MEM (300 µl per well of the 6-well plate, Gibco) and plasmid DNA (for the amounts see Table 4).

**Table 4:** Transfection Scheme for PEI transfection in step 1.2.

Vector	Amount (µg)
pBob-eGFP-LC3B	1.75
pMDLg	0.87
RSV-Rev	0.44
pmD.G	0.5
  1.2.4. Combine Mix A and Mix B and incubate at RT for 20 min.
  1.2.5. Add the transfection mix dropwise onto the cells without disturbing the monolayer.
  1.2.6. Change the medium to DMEM + 3 (2.5% FBS) 6 h post-transfection.
  1.2.7. 24 h post-transfection, the cells should exhibit bright green fluorescence. Repeat if no fluorescence is observed.
  1.2.8. Harvest the SN 48 h post transfection and clear it by centrifugation (2,700 g, 10 min, 4 °C) and filtering (Millex-HA Filter, 0.45 µm, Merck).
1.3. Transduce 5 × 10^5^ cells of the selected target cell line (e.g. HEK293T, HeLa) with 100 µl of the SN harvested in step 1.2.8.
1.4. Two days post-transduction, detach the cells using Trypsin (0.05%, GIBCO, for adherent cells), pellet at 300 g, 3 min, 4 °C.
1.5. Remove the SN and replace it with fresh medium without FBS to a concentration of 5–10 × 10^6^ cells per ml in a 15 ml Falcon tube. Filter through round-bottom tubes with a cell strainer cap (5 ml, Falcon). Sort samples on a FACS sorter e.g. BD FACSAria III, using a 100-micron ceramic nozzle. The flow rate was kept below 2, and the purity setting was set to a single cell. Sort one cell per 96-well F-bottom well.
  **Important:** The nozzle size needs to be larger than the cell passing through it. Avoid crushing or stressing the cells too much.
1.6. Provide single cells with medium supplemented with 20% FBS according to the cell type. Select eGFP-LC3B cells, with moderate eGFP MFIs (Fig. 1B).

**Figure 1:**
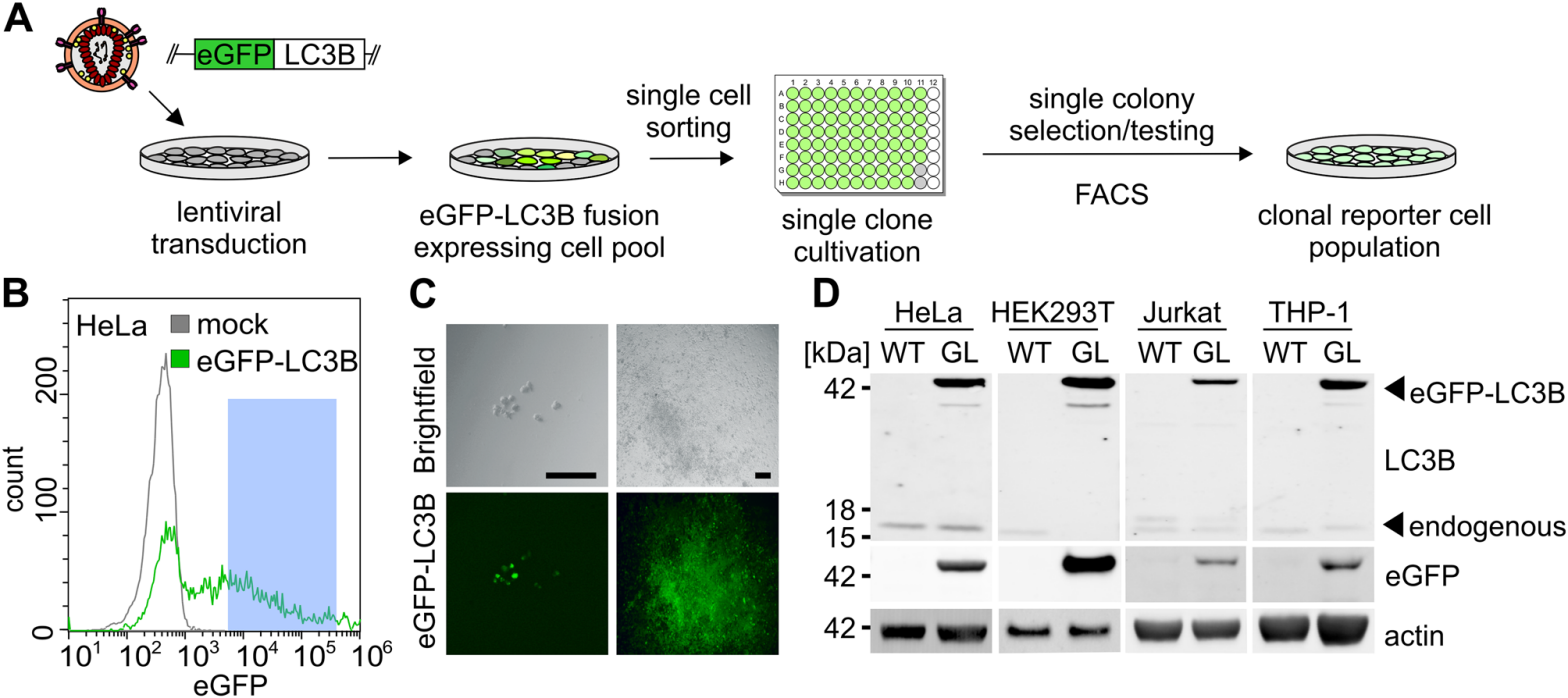
Construction of autophagy reporter cell lines. (**A**) Schematic overview. After random genomic integration of an eGFP-LC3B expressing cassettes into target cells by lentiviral delivery, single clones are isolated via FACS sorting and grown into clonal cell lines. (**B**) FACS sorting gating strategy for eGFP-LC3B-lentivirus transduced HeLa cells. Viable cell clones with intermediate eGFP-LC3B expression were chosen (blue). (**C**) Exemplary images of HeLa single cell clones expressing eGFP-LC3B in 96-well plates. Left panels: brightfield image and corresponding eGFP fluorescence (green) of a single clone after 1 week. Right panel, brightfield image and corresponding eGFP fluorescence (green) after 3 weeks, before transfer into larger wells. Size marker, 100 µm. (**D**) Detection of eGFP-LC3B expression in isolated clonal HEK293T, HeLa, Jurkat and THP-1 cell lines, comparing them to their respective parental cell line. Immunoblotting of whole cell lysates using anti-GFP, anti-LC3B and anti-β-actin antibodies. Uncropped western blots in Supplementary Fig. 3.
  **Important**: Some cell lines do not grow efficiently from single clones. Avoid isolating cell lines expressing too much or no eGFP-LC3B.
1.7. Approximately 3–6 weeks post sorting, clones can be transferred from half-confluent 96-wells directly into 12-well plates. Avoid overgrowing in 96-wells or at any step.
1.8. eGFP-LC3B expression and fluorescence can be monitored using a fluorescence microscope and clones with little to no detectable fluorescence are discarded. Clones with clearly visible aggregations of eGFP-LC3B in the cytoplasm are also discarded.
1.9. Grow clones up to confluent T25 flasks and then proceed to verify the clones.
1.10. Confirm whether the cell clones contain the reporter:
  1.10.1. Generate whole-cell lysates of the cell line (as described in the short Methods section), and immunoblot with anti-GFP antibody and anti-LC3B antibody. An additional band at around 50 kDa, representing the fusion protein, should appear in the stable cell lines compared to their parental cell lines.
    **Note**: In some cell lines an additional band at the height of eGFP (ca. 27 kDa) may appear due to the processing of eGFP-LC3B. We have not observed any negative impact of this additional eGFP band on the sensitivity and responsiveness of the respective cell lines. Cell lines with further degradation products should be discarded.
  1.10.2. Fix the cells using 4% PFA on glass slides for confocal microscopy (as described in the short methods section). Take confocal images and analyze the number of autophagosomes upon treatment with positive controls such as Rapamycin (1 µM) or Chloroquine (10 µM) for 4 h. The number of autophagosomes (= eGFP-LC3B) puncta per cell should significantly increase compared to mock-treated cells.
  1.10.3. Follow the protocol for high-throughput autophagy quantification to verify that the cell line is suitable for the approach and contains enough eGFP-LC3B for cytoplasmic washout and subsequent analysis. Use treatment with Rapamycin (1 µM) or Chloroquine (10 µM) for 4 h and compare to cells treated with the carrier (e.g. Dimethyl sulfoxide (DMSO) or water).
    **Important**: The level of expressed eGFP-LC3B impacts the sensitivity of the assay. Generally, we have found that a medium amount of eGFP-LC3B seems to be optimal. Too low eGFP-LC3B expression causes a loss of detectable eGFP-LC3B fluorescence after washout. Too high eGFP-LC3B causes a high baseline of autophagosome associated eGFP-LC3B, as well as occasionally aggregates observed in confocal microscopy.
    Produce frozen stocks as early as possible to preserve the generated cell line at a passage number as low as possible.

### High-throughput quantification of autophagy

A detailed list of materials is provided in Table 5.

**Table 5:** Materials used for high throughput quantification of autophagy.

Product	Source	Product number
InSolution Rapamycin	Merck	553211
Chloroquine diphosphate salt	Santa Cruz Biotechnology	sc-205629
Bafilomycin A1	Santa Cruz Biotechnology	sc-201550
Dimethyl sulfoxide	Merck	102952
PBS, no calcium, no magnesium	Gibco	14190094
Trypsin 0.05%/EDTA 0.02% in PBS, w/o: Ca and Mg	PAN-Biotech	P10-023100
Saponin	Sigma-Aldrich	47036
Paraformaldehyde solution 4% in PBS	Santa Cruz Biotechnology	sc-281692
**Media**		
RPMI 1640 Medium	Gibco	21875034
Gibco DMEM, high glucose, no glutamine	Gibco	11500416
Fetal Bovine Serum, qualified, Brazil	Gibco	10270106
Penicillin–Streptomycin, 10,000 U/ml Penicillin, 10 mg/ml Streptomycin	PAN-Biotech	P06-07050
L-Glutamine 200 mM	PAN-Biotech	P04-80050

2.1. Grow cells in either F-bottom (adherent cell types) or V-bottom (suspension cell types) 96-well plates. Seeding of 50,000 cells per well for adherent cells and 100,000 cells per well for non-adherent cells is recommended, 18 h before a 4-h assay.
  **Important**: At the time of harvest, the cells should be approximately confluent, and kept in fully supplemented medium which is still at optimal pH. Adjust the cell number/density at the time of seeding to meet these criteria. Do not change medium/nutrient conditions before treatment/harvesting. Any stress on the cells impacts autophagy and thus may bias the assay. Consider appropriate negative and positive controls. Established drugs like Rapamycin, Chloroquine, or Bafilomycin A1 are recommended as controls. Negative controls should be treated the same way as the samples, e.g. add the carrier for a drug in the same concentration to the mock SN. Screening results have to be confirmed using orthogonal methods for assessing autophagy levels.
2.2. For adherent cells: discard the SN without disturbing the monolayer.
  for suspension cells: Spin down the cells at 500 g, 3 min, 4 °C, discard the SN.
2.3. Wash the treated (or transfected or infected) cells with 100 µl 1xPBS (Gibco).
  **Important**: Proceed with the harvesting as quickly as possible until the cells are in saponin-containing PBS (step 2.6)!
2.4. Only for adherent cells: Transfer cells into V-bottom 96-well plate.
  2.4.1. Discard the SN without disturbing the monolayer.
  2.4.2. Detach cells with 50 µl Trypsin (0.05%, PAN-Biotech), incubate for 5 min at 37 °C.
  2.4.3. Transfer cells to a V-bottom 96-well plate.
  2.4.4. Add 50 µl DMEM + 3 to the original F-bottom 96-well plate, transfer the medium to the same V-bottom 96-well plate as in the previous step.
2.5. Spin down the cells at 500 g, 3 min, 4 °C, discard the SN.
2.6. Add 100 µl 0.05% saponin in 1xPBS.
2.7. Resuspend the cells in the plate by shaking or pipetting up and down.
2.8. Incubate for at least 20 min (**crucial!**) at 4 °C.
  **Important**: Incubation longer than 40 min may lead to full lysis of the cells.
2.9. Spin down the cells at 500 g, 3 min, 4 °C, discard SN.
2.10. Wash cells at least twice (**!**) with 100 µl 1xPBS.
  **Important**: Additional washing steps increase the signal/noise ratio. However, at a loss of cell count. We have found that washing twice is sufficient for most applications, and cell numbers stay within a reasonable range.
2.11. Spin down the cells at 500 g, 3 min, 4 °C, discard SN.
2.12. Use one of three different subsequent treatments (see Fig. 3D for comparison).
  2.12.1. No fixation. Cells are left unfixed after saponin treatment.
    **Important**: Cells disintegrate rapidly if they are not fixed after saponin treatment, however, the cells are good for FACS analysis for up to 3 h following the treatment if extensively washed.
  2.12.2. Paraformaldehyde (PFA) fixation:
    2.12.2.1. Spin down at 500 g, 3 min, 4 °C, discard SN.
    2.12.2.2. Add 100 µl 1% PFA in 1xPBS (Santa Cruz).
    2.12.2.3. Incubate for 20 min at RT in the dark.
    2.12.2.4. Spin down at 500 g, 3 min, 4 °C, discard SN.
    2.12.2.5. Add 100 µl 1xPBS (Gibco).
      **Important:** Fixed cells can be stored in the fridge (4 °C) for analysis up to a week. But fluorescence of the cells wanes over time. Analyze as early as possible.
  2.12.3. Methanol fixation:
    2.12.3.1. Spin down 500 g, 3 min, 4 °C, discard SN.
    2.12.3.2. Add 100 µl -20 °C cold 100% methanol (MeOH) (Merck) per well.
    2.12.3.3. Incubate 20 min at -20 °C.
    2.12.3.4. Spin down 500 g, 3 min, 4 °C, discard SN.
      **Important**: Do not let the MeOH on the cells warm up to RT!
    2.12.3.5. Add 100 µl ice-cold 1xPBS.
    2.12.3.6. Spin down 500 g, 3 min, 4 °C, discard SN.
      **Important**: Get rid of traces of MeOH for downstream analyses.
    2.12.3.7. Add 100 µl 1xPBS.
      **Important**: Fixed cells can be stored in the fridge (4 °C) for analysis up to a week.
      But fluorescence of the cells wanes over time. Analyze as early as possible.
2.13. Proceed to flow cytometry. Note that saponin-treated cells appear smaller than non-treated cells and have less eGFP content, as well as less autofluorescence, see Fig. 3B.
2.14. Use the voltage or gain settings of the cytometer to set the MFI of mock-treated cells to 1,000.
2.15. Living and single cells are gated using FSC and SSC, measure at least 10,000 living/single-cell events.
  **Important**: Quantification using less than 10,000 cells still is possible, however, the inherent heterogeneity of autophagy, even within cell populations derived from a single clone, may result in higher deviations between biological replicates.
2.16. Extract the MFI of eGFP-LC3B from all samples. For background correction, the MFI measured in mock conditions is subtracted from the MFI of treated samples (see Flow Cytometry and Cell Sorting).

### Applications of high-throughput quantification of autophagy (Examples)

A detailed list of materials is provided in Table 6.

**Table 6:** Materials used for application examples of the eGFP-LC3B reporter system.

Product	Source	Product number
InSolution Rapamycin	Merck	553211
Chloroquine diphosphate salt	Santa Cruz Biotechnology	sc-205629
Bafilomycin A1	Santa Cruz Biotechnology	sc-201550
Dimethyl sulfoxide	Merck	102952
Polyethylenimine, branched	Sigma-Aldrich	408727
Opti-MEM I Reduced Serum Medium	Gibco	31985047
IAV, PR8	ATCC	VR-95
EMCV, EMC	ATCC	VR-129B
MeV, Schwarz strain	K.-K. Conzelmann	^40^
Paraformaldehyde solution 4% in PBS	Santa Cruz Biotechnology	sc-281692
Amino Acids	Sigma	various
Methanol	Merck	32213
Human CRISPR Knockout Pooled library (GeCKO v2) (39)	Addgene	#1000000048
NEBNext High-Fidelity 2X PCR Master Mix	New England Biolabs	M0541
Monarch PCR & DNA Cleanup Kit	New England Biolabs	T1030
UltraPure DNase/RNase-Free Distilled Water	Invitrogen	10977
Quick-DNA Midiprep Plus Kit	Zymo Research	D4075
Puromycin Dihydrochloride	Gibco	A1113803
PBS, no calcium, no magnesium	Gibco	14190094
Falcon Round-Bottom Tubes with Cell Strainer Cap, 5 mL	Falcon	38030
Saponin	Sigma-Aldrich	47036
**Media**		
RPMI 1640 Medium	Gibco	21875034
Gibco DMEM, high glucose, no glutamine	Gibco	11500416
Fetal Bovine Serum, qualified, Brazil	Gibco	10270106
Penicillin–Streptomycin, 10,000 U/ml Penicillin, 10 mg/ml Streptomycin	PAN-Biotech	P06-07050
l-Glutamine 200 mM	PAN-Biotech	P04-80050

***Important: ***All hits in screening approaches have to be verified using orthogonal methods to assess autophagy (for a comprehensive overview of available methods see^24^).

3.1. High-throughput screening for novel autophagy modulating drugs.
  3.1.1. Seed eGFP-LC3B reporter cells in 96-well plates, 50,000 cells per well in F-bottom plates for adherent cells, and 100,000 cells per well in V-bottom plates for non-adherent cells. The total amount of medium should be 90 µl fully supplemented medium (e.g. DMEM + 3) per well.
  3.1.2. Grow adherent cells overnight at 37 °C, 5% CO_2_, 90% humidity. Seed non-adherent cells directly before applying the treatment.
    **Important**: Do not seed cells from overgrown flasks or nutrient-deprived cell stocks.
  3.1.3. Add 10 µl of treatment solution to the cells. Several examples of drug treatment were used to verify the generated cell lines. As a control, use the same amount of carrier liquid (e.g. water or DMSO).
    **Important**: Do not exchange the medium before treatment. Do not vary the volume between treatments and do not add additional medium, FBS, or salt (beyond 150 mM). Any stress may cause changes in autophagic flux and obscure effects of drugs, thus treat your cells carefully.
    For our exemplary experiments (Fig. 4) we used:
    3.1.3.1. Rapamycin (InSolution Rapamycin, Merck), which induces autophagy by inhibiting the mTORC1 complex^6,43^, was used in a range of 2 µM to 15 nM (Fig. 4).
    3.1.3.2. Chloroquine (Chloroquine diphosphate, Santa Cruz Biotechnology), which blocks the turnover of autophagosomes^44–46^, was used in a range of 20 µM to 150 nM (Fig. 4).
    3.1.3.3. Bafilomycin A1 (Santa Cruz Biotechnology), which blocks the turnover of autophagosomes^47,48^, was used in a range of 625 nM to 5 nM (Fig. 4).
    3.1.3.4. Amino acids (Sigma) dissolved in water were used at a final concentration of 1 mg/ml. (Fig. 4E).
  3.1.4. Harvest and analyze the cells 4 h post-treatment as described in the basic protocol.
    **Optionally**: Cells can be treated longer or shorter, we have observed that a time frame between 2 and 4 h is optimal for most drug-based applications. However, drugs that are expected to block autophagic flux may be kept on the cells for longer to measure the accumulation of vesicles from basal autophagy. Please note that especially blocking autophagy decreases the viability of the cells after 4–6 h.
3.2. Manipulation of autophagy by viruses.
  3.2.1. Seed eGFP-LC3B reporter cells in 96-well plates, 50,000 cells per well in F-bottom plates for adherent cells, and 100,000 cells per well in V-bottom plates for non-adherent cells. Seed cells in 90 µl fully supplemented medium (e.g. DMEM + 3) or infection medium per well.
    **Important**: Control medium changes with parallel medium changes in mock-infected wells.
  3.2.2. Infect cells by addition of 10 µl virus-containing solution (or mock carrier) with an appropriate MOI at different time points, e.g. as indicated in Fig. 5.
    **Important:** Add the same type/amount of fluid (lacking virus) used for infection to the medium of the mock wells. E.g. if the virus was produced in Vero cells, add an equal amount SN from non-infected Vero cells to the mock wells. Alternatively, use the buffer the virus is stored in, e.g. after purification.
  3.2.3. Harvest cells and analyze as described in the basic protocol.
    **Important**: Discard virus-containing SN according to your biosafety protocols. Keep in mind that some viruses like EMCV are not inactivated by saponin-treatment.
3.3. Identification of novel autophagy regulating factors using overexpression
  3.3.1. Seed 30,000 eGFP-LC3B HEK293T reporter cells in F-bottom 96-well plates in 100 µl DMEM + 3 per well.
  3.3.2. Transfect vectors coding the proteins of interest 18 h post-seeding and the empty vector control using PEI and a maximum of 0.15 µg of DNA per well.
    3.3.2.1. Mix A: Mix Opti-MEM (10 µl per well of the 96-well plate) and PEI (2 µl PEI per µg DNA) and incubate for 5 min at RT.
    3.3.2.2. Mix B: Mix Opti-MEM (10 µl per well of the 96-well plate) and DNA (0.15 µg per well of the 96-well) and add the same volume of Mix A.
    3.3.2.3. Incubate the transfection mix for 20 min at RT.
    3.3.2.4. Add the transfection mix dropwise onto the cells without disturbing the monolayer.
      **Important**: Transfection induces autophagy. We have tested a few transfection reagents and found that PEI and calcium phosphate transfection induce the lowest amount of autophagy as opposed to commercially available transfection reagents. We generally use PEI, as it results in higher transfection rates/expression rates than calcium phosphate transfection. Thus, pre-test your transfection method. Alternatively, transduction can be used, however, gene expression is lower and an autophagic response is still induced albeit to a lesser degree.
  3.3.3. Change medium to DMEM + 3 4–6 h post-transfection.
  3.3.4. 48 h post-transfection, cells are harvested and analyzed as described in the basic protocol.
3.4. Identification of novel autophagy regulating factors using CRISPR/Cas9.
  **Important**: This procedure was modified from Joung et al^49^. Please consult the original publication for a comprehensive protocol. Create/Amplify/Determine the titer of the Human CRISPR Knockout Pooled Library (GeCKO v2)^37^ according to^49^.
  3.4.1. Transduce 1 × 10^9^ autophagy reporter cells (e.g. Jurkat eGFP-LC3B) with an MOI of 0.3.
  3.4.2. 24 h post-infection, select with appropriate concentrations of puromycin (ranging from 0.25 µg/ml to 1 µg/ml, e.g. 0.75 µg/ml for Jurkat eGFP-LC3B).
  3.4.3. Add fresh, fully supplemented medium to the cells every two days.
    **Important**: Do not let the cells starve or overgrow.
  3.4.4. 10 days post-infection, harvest the cells by pelleting (300 g, 5 min, 4 °C).
  3.4.5. Determine the initial cell number for the protocol. It should range between 1 × 10^10^ and 1 × 10^11^.
  3.4.6. Take 1 × 10^8^ cells from the ‘sample’ as ‘input’ for the CRISPR screen.
  3.4.7. Process the ‘sample’ and ‘input’ with saponin according to the basic protocol.
  3.4.8. Fix cells in MeOH (**!**) according to the basic protocol.
  3.4.9. Take up the cells in 10 ml 1xPBS and filter through a round-bottom tube with a cell strainer cap (5 ml, Falcon).
  3.4.10. Sort the ‘sample’ cells using a FACS sorter into high, medium, and low autophagosome content cells (see Fig. 5D). Sort at least 5 × 10^7^ cells for each fraction. Discard the medium autophagy inducing cells.
    **Important**: We usually get about 8–10% of the total cell number for the high eGFP-LC3B fluorescence cells and 8–10% of the total cell number for the low eGFP-LC3B fluorescence cells. In cells transduced with non-targeting CRISPR vector, less than 1% of the total cell number should fall into the low or high category. Sorting of the cells takes a few hours (~ 8–10) depending on the flow rate. Adjust the flow rate and the concentration of the treated sample to the highest possible flow rates.
  3.4.11. Pellet the sorted and input cells (300 g, 5 min, 4 °C).
  3.4.12. Use the Zymo Research Quick-gDNA MidiPrep Kit to extract the genomic DNA from the cells (according to the manufacturer's protocol).
  3.4.13. Elute the genomic DNA in 200 µl Pyrogen/RNase/DNase free water and measure its concentration.
    **Important**: The concentration may be very low (< 10 ng/µl) depending on the number of sorted cells. The DNA may be concentrated using an 80% isopropanol precipitation.
  3.4.14. Use PCR to amplify the genomically integrated sgRNAs, use individual barcodes for ‘input’, ‘sample-high’, and ‘sample-low’.
    Set up 10 PCR reactions according to Tables 7, 8, and 9.

**Table 7:** Pipetting scheme for sgRNA amplification PCR.

Component	Amount per reaction (µl)	Final concentration
NEBNext High Fidelity PCR Master Mix, 2 ×	25	1x
Pooled sgRNA library template		10.4 ng/µl
NGS-Lib-Fwd primer (unique), see Table 9	1.25	0.25 µM
NGS-Lib-KO-Rev, see Table 9	1.25	0.25 µM
Pyrogen/RNase/DNase free water		add to a total volume of 50 µl

**Table 8:** PCR protocol.

Step	Cycle #	Temperature (°C)	Duration
Initial denaturization	1	98	3 min
Denaturization	2–29	98	10 s
Primer annealing		63	10 s
Extension		72	25 s
Final extension	30	72	2 min

**Table 9:** Primer sequences.

NGS-Lib-Fwd-1	AATGATACGGCGACCACCGAGATCTACACTCTTTCCCTACACGACGCTCTTCCGATCTTAAGTAGAGGCTTTATATATCTTGTGGAAAGGACGAAACACC
NGS-Lib-Fwd-2	AATGATACGGCGACCACCGAGATCTACACTCTTTCCCTACACGACGCTCTTCCGATCTATCATGCTTAGCTTTATATATCTTGTGGAAAGGACGAAACACC
NGS-Lib-Fwd-3	AATGATACGGCGACCACCGAGATCTACACTCTTTCCCTACACGACGCTCTTCCGATCTGATGCACATCTGCTTTATATATCTTGTGGAAAGGACGAAACACC
NGS-Lib-Fwd-4	AATGATACGGCGACCACCGAGATCTACACTCTTTCCCTACACGACGCTCTTCCGATCTCGATTGCTCGACGCTTTATATATCTTGTGGAAAGGACGAAACACC
NGS-Lib-Fwd-5	AATGATACGGCGACCACCGAGATCTACACTCTTTCCCTACACGACGCTCTTCCGATCTTCGATAGCAATTCGCTTTATATATCTTGTGGAAAGGACGAAACACC
NGS-Lib-Fwd-6	AATGATACGGCGACCACCGAGATCTACACTCTTTCCCTACACGACGCTCTTCCGATCTATCGATAGTTGCTTGCTTTATATATCTTGTGGAAAGGACGAAACACC
NGS-Lib-Fwd-7	AATGATACGGCGACCACCGAGATCTACACTCTTTCCCTACACGACGCTCTTCCGATCTGATCGATCCAGTTAGGCTTTATATATCTTGTGGAAAGGACGAAACACC
NGS-Lib-Fwd-8	AATGATACGGCGACCACCGAGATCTACACTCTTTCCCTACACGACGCTCTTCCGATCTCGATCGATTTGAGCCTGCTTTATATATCTTGTGGAAAGGACGAAACACC
NGS-Lib-Fwd-9	AATGATACGGCGACCACCGAGATCTACACTCTTTCCCTACACGACGCTCTTCCGATCTACGATCGATACACGATCGCTTTATATATCTTGTGGAAAGGACGAAACACC
NGS-Lib-Fwd-10	AATGATACGGCGACCACCGAGATCTACACTCTTTCCCTACACGACGCTCTTCCGATCTTACGATCGATGGTCCAGAGCTTTATATATCTTGTGGAAAGGACGAAACACC
NGS-Lib-KO-Rev-1	CAAGCAGAAGACGGCATACGAGATTCGCCTTGGTGACTGGAGTTCAGACGTGTGCTCTTCCGATCTCCGACTCGGTGCCACTTTTTCAA
NGS-Lib-KO-Rev-2	CAAGCAGAAGACGGCATACGAGATATAGCGTCGTGACTGGAGTTCAGACGTGTGCTCTTCCGATCTCCGACTCGGTGCCACTTTTTCAA
NGS-Lib-KO-Rev-3	CAAGCAGAAGACGGCATACGAGATGAAGAAGTGTGACTGGAGTTCAGACGTGTGCTCTTCCGATCTCCGACTCGGTGCCACTTTTTCAA
  3.4.15. A single band at 260–270 bp should appear on an agarose gel.
  3.4.16. Combine 2 PCR reactions each for one NEB Monarch PCR & DNA Cleanup Kit (5 µg) column according to the manufacturer’s instructions (e.g. 5 purifications per sample).
  3.4.17. Elute in 10 µl Pyrogen/RNase/DNase free water.
  3.4.18. Pool the extracted DNA to have three samples: ‘input’, ‘high’, and ‘low’. Proceed with next-generation-sequencing.

## Results

### Construction of eGFP-LC3B expressing cell lines

**Figure 2:**
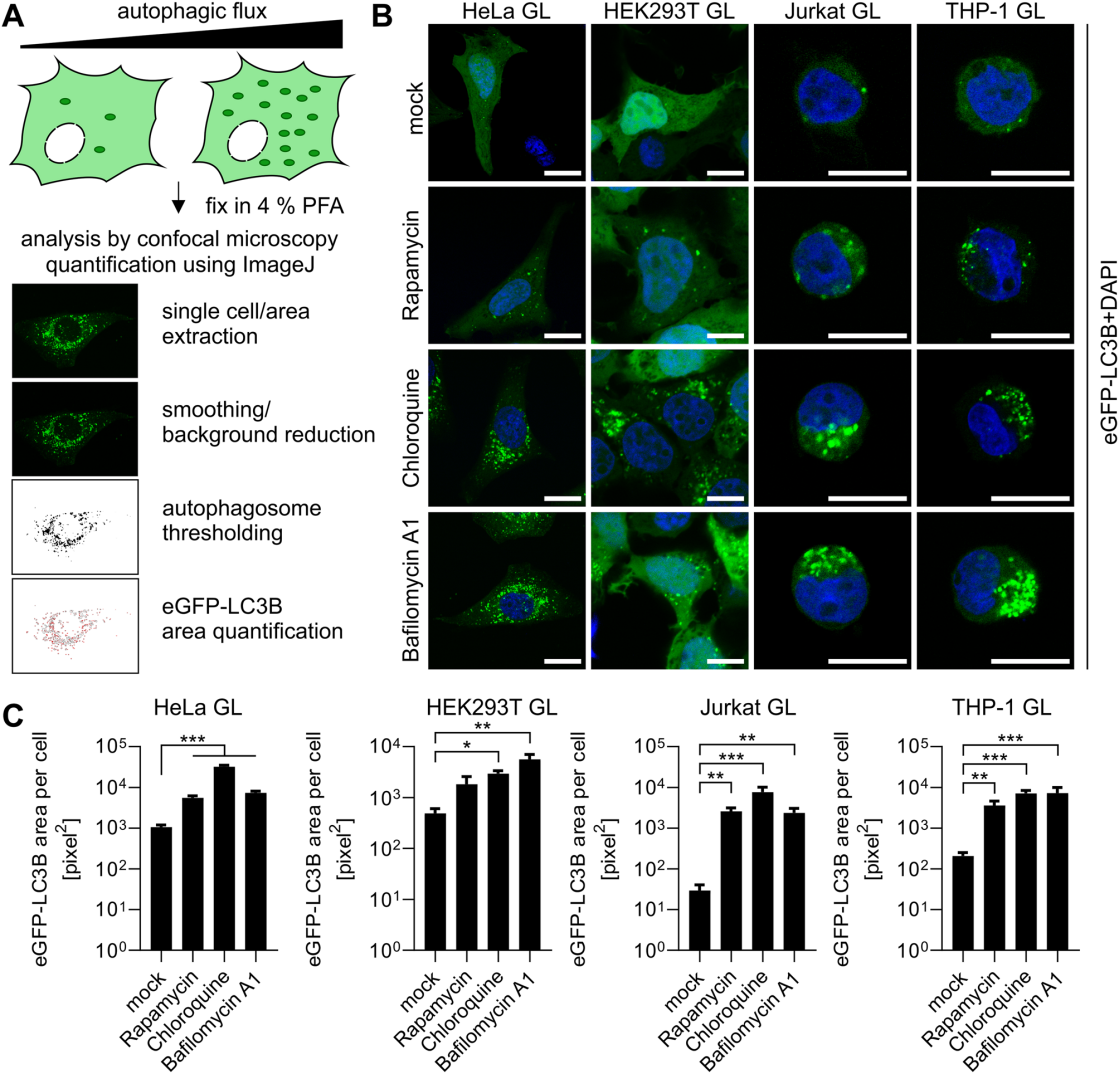
Quantification of eGFP-LC3B + vesicles using confocal fluorescence microscopy. (**A**) Schematic overview of the method. eGFP-LC3B expressing cell lines are fixed in 4% PFA and images taken by confocal fluorescence microscopy. Using ImageJ, the area and the count of eGFP-LC3B puncta in the cells can be quantified to measure autophagy. (**B**) Exemplary confocal images of eGFP-LC3B (green) expressing HeLa, HEK293T, Jurkat, and THP-1 cells. The cells were either mock-treated or treated with Rapamycin (1 µM), Chloroquine (10 µM), or Bafilomycin A1 (2.5 µM) for 4 h before fixation. Nuclei, DAPI (blue). Size marker, 10 µm. (**C**) Quantification of the eGFP area per cell of HeLa eGFP-LC3B cells, HEK293T eGFP-LC3B, Jurkat eGFP-LC3B, or THP-1 eGFP-LC3B either mock-treated or treated with Rapamycin (1 µM), Chloroquine (10 µM), or Bafilomycin A1 (2.5 µM) for 4 h before fixation, using the method described in **A**. Results are shown as mean(HeLa GL n = 50–100, HEK293T GL n = 5–6, Jurkat GL n = 6–11, and THP-1 GL n = 10–12) ± SEM, statistical significance was assessed using one-way ANOVA. *p ≤ 0.05, **p ≤ 0.01, ***p ≤ 0.001.

Highly robust quantification of autophagy can be achieved by measuring the amount of LC3B-positive vesicles, a hallmark of autophagy, using eGFP-LC3B expressing reporter cells^24,33^. To this end, we constructed autophagy reporter cell lines stably expressing eGFP-LC3B from a genomically integrated, CMV-promoter controlled, expression cassette. Third generation lentiviral particles harboring the expression cassette were generated and target cell lines transduced. The cell lines include cell lines (HeLa) classically used for autophagy research, easy-to-transfect cell lines (HEK293T) and cells of the immune system like Monocyte-like and T cells (THP-1 and Jurkat) (Fig. 1A). Following transduction, single-cell clones were sorted from a pool of medium level eGFP-LC3B expressing cells (exemplarily shown for HeLa eGFP-LC3B cells in Fig. 1B). These single-cell clones were grown into clonal cell lines and eGFP-LC3B fluorescence monitored by fluorescence microscopy (Fig. 1C). Unchanged p62 levels between parental and stable cell lines suggest that autophagic flux was not significantly altered by the expression of eGFP-LC3B (Fig. S1A). Finally, expression and size of the fusion protein eGFP-LC3B (ca. 45 kDa) was confirmed by immunoblotting (Fig. 1D) for all reporter cell lines and compared to their respective parental cell lines: HeLa, HEK293T, Jurkat, and THP-1. All reporter cell lines (Fig. 2A, B) displayed the characteristic puncta of eGFP-LC3B-positive autophagosomes in addition to a diffuse cytoplasmic signal. To confirm that the reporter cell lines respond to stimulation and blockage of autophagy, they were treated with different drugs that induce (Rapamyicin) or block autophagic flux (Chloroquine, Bafilomycin A1). Whereas mock-treated cells show a low number of autophagosomes (= eGFP-LC3B puncta in the cytoplasm), the number of puncta visibly increased upon Rapamycin treatment. Chloroquine and Bafilomycin A1 treatment led to an accumulation of perinuclear autophagosomes (Fig. 2B). The pixel area of eGFP-LC3B, which correlates with autophagy levels, was quantified using semi-automated analysis (Fig. 2C). Confocal images were taken and randomly selected single cells extracted. Aided by an ImageJ macro^50^, automatic thresholding, and particle counting resulted in the pixel area of autophagosomes (Fig. 2A, C). Western blotting confirmed that p62 accumulates in the reporter cell lines upon Bafilomycin A1 treatment, demonstrating functional autophagy (Fig. S1B). Taken together, the generation of eGFP-LC3B expressing autophagy reporter cell lines derived from HeLa, HEK293T, Jurkat, and THP-1 cells was successful.

### Rapid quantification of autophagosomes using eGFP-LC3B expressing cells

**Figure 3:**
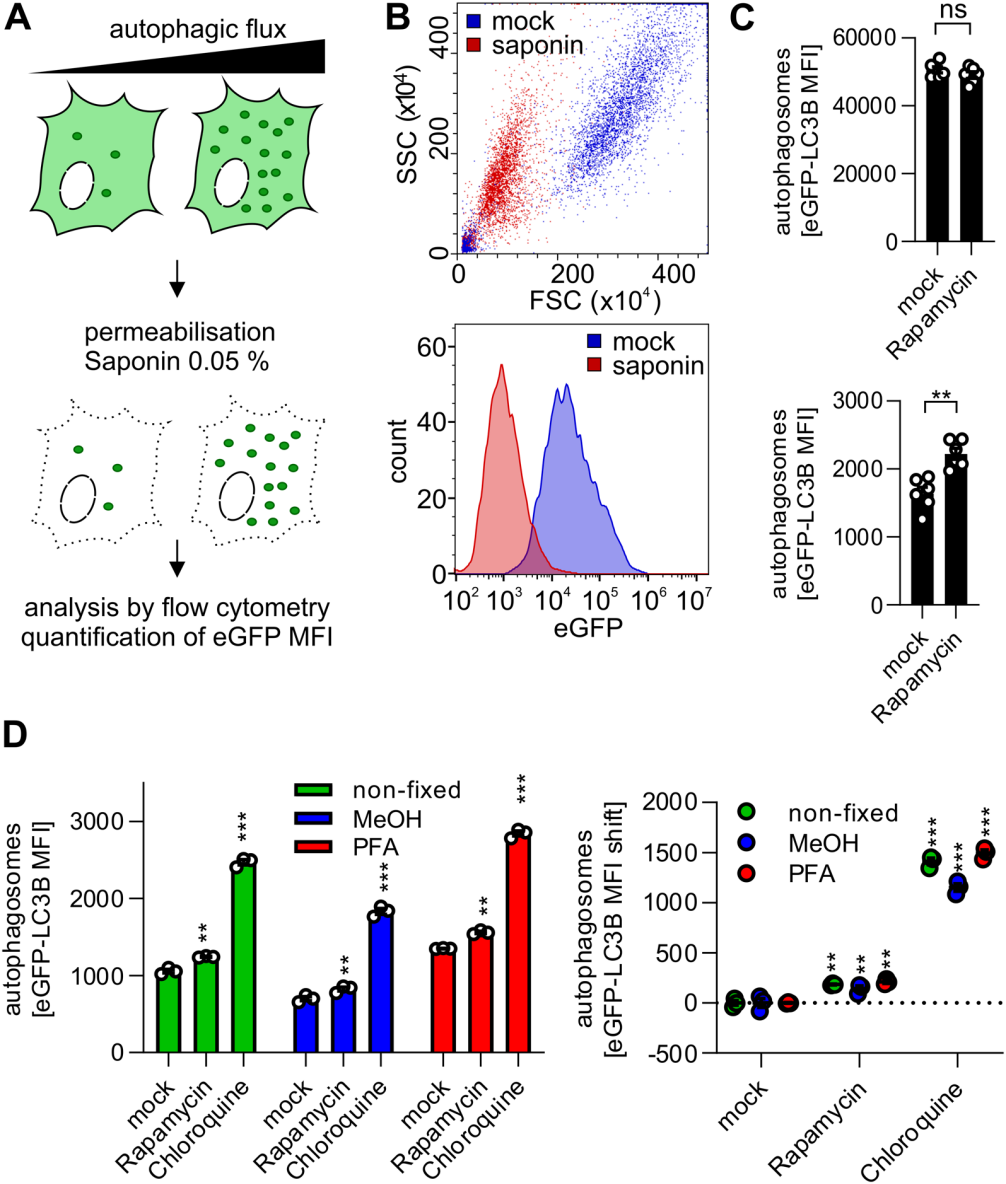
Quantification of eGFP-LC3B + vesicles using high throughput flow cytometry. (**A**), Schematic overview of the method. eGFP-LC3B expressing cell lines are permeabilized with 0.05% saponin containing buffer, the non-membrane bound eGFP-LC3B is washed out, followed by fixation with 4% PFA. Using FACS, the amount of membrane-bound eGFP-LC3B per cell is quantified as eGFP mean fluorescent intensity (MFI). (**B**), Forward and side scatter of HeLa eGFP-LC3B cells in flow cytometry before (blue) and after saponin treatment (red) (top panel). eGFP-LC3B fluorescence is decreased after saponin treatment of HeLa eGFP-LC3B cells (bottom panel). (**C**), Total eGFP-LC3B MFI of HeLa eGFP-LC3B cells, either mock-treated or treated with Rapamycin (2 µM, 4 h) (top panel). eGFP-LC3B MFI of HeLa eGFP-LC3B cells, either mock-treated or treated with Rapamycin (2 µM, 4 h) after saponin permeabilization and cytoplasmic washout of the cells (bottom panel). Data are shown as mean (n = 6) ± SEM. (**D**), HeLa eGFP-LC3B cells were either mock-treated or treated with, Chloroquine (10 µM) or Rapamycin (1 µM) for 4 h and subsequently saponin treated. eGFP MFIs were quantified, either in non-fixed (green), methanol fixed (blue), or paraformaldehyde (PFA) fixed cells (red). The left panel shows the raw MFI, the right panel shows the background adjusted values. Data are shown as mean (n = 3) ± SEM. Statistical significance was assessed using unpaired t-tests (C) or one-way ANOVA (D). ns = not significant, **p ≤ 0.01, ***p ≤ 0.001.

For high-throughput applications, an efficient system to quantify LC3B-positive autophagosomes is desirable. To measure autophagy using flow cytometry, eGFP-LC3B-II decorated autophagosomes have to be separated from cytosolic eGFP-LC3B-I. Thus, eGFP-LC3B expressing cells were permeabilized with 0.05% saponin containing PBS, the cytoplasm subsequently washed out (Fig. 3A) and only autophagosome-bound eGFP-LC3B-II retained inside the cells^24,33^. As determined by flow cytometry, the cells decreased in size and granularity (Fig. 3B, upper panel). Complete permeabilization as indicated by cell size is reached after 10 min (Fig. S1C). Furthermore, after successful removal of cytoplasmic eGFP-LC3B-I, the mean eGFP fluorescence levels are drastically reduced (Fig. S1D), representing only fluorescence of autophagosome-bound eGFP-LC3B-II (Fig. 3B, lower panel). Therefore, treatment with saponin allows quantification of changes in autophagosome numbers, which are not visible in non-permeabilized cells (Fig. 3C). The washout procedure is very robust, and only minor variations are observed in the treatment (Fig. S1E). It is possible to fix the cells to preserve the eGFP-LC3B signal after saponin permeabilization for longer storage using two different methods: PFA and MeOH. Compared to non-fixed cells, the signal in PFA-fixed cells was well preserved, MeOH fixation, however, caused a slight drop of the absolute eGFP-LC3B signal (Fig. 3D). However, as the differences between differently treated cells were preserved, both fixation methods were suitable. Thus, isolation of autophagosome-bound eGFP-LC3B and its detection using flow cytometry was successful and can be used to reveal changes in autophagy levels.

### High-throughput discovery of compounds that modulate autophagy

**Figure 4:**
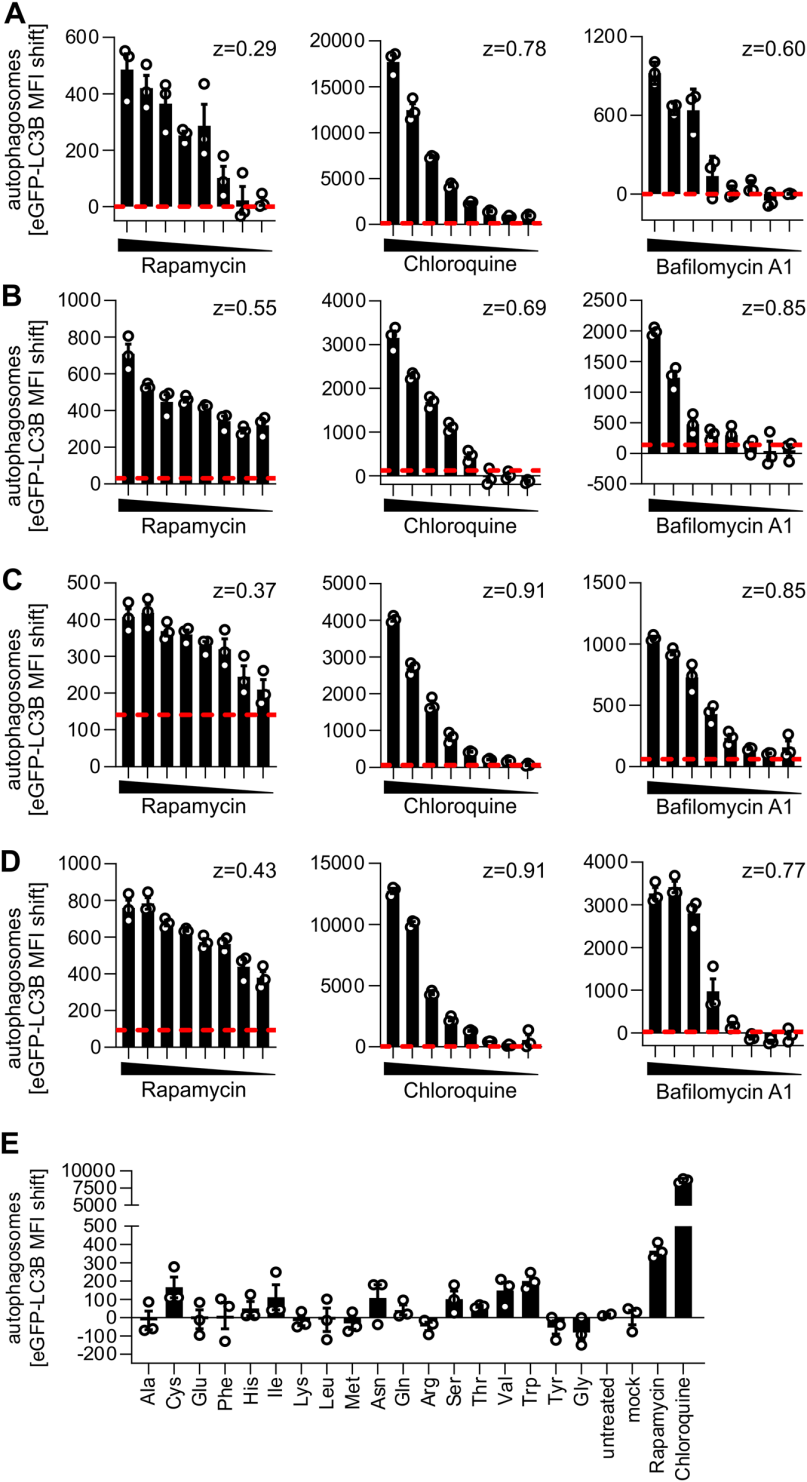
Analysis of autophagy-modulating compounds using eGFP-LC3B expressing cell lines. The eGFP-LC3B MFI of saponin-treated eGFP-LC3B expressing HeLa (**A**), HEK293T (**B**), Jurkat (**C**), or THP-1 (**D**) cells was quantified using flow cytometry and the background (= mock) subtracted. Cells were either mock-treated or treated with Rapamycin (2 µM—15 nM; left panel), Chloroquine (20 µM–150 nM; middle panel), or Bafilomycin A1 (625 nM–5 nM, right panel) for 4 h. Z-factors are indicated next to the respective diagram. The red dotted line indicates the double standard deviation of the mock control. Data are shown as mean(n = 3) ± SEM. (E) Background subtracted eGFP-LC3B MFIs of HeLa GL cells either mock-treated, or treated with 18 different human amino acids (1 mg/ml, as indicated in three-letter code), Rapamycin (1 µM), or Chloroquine (10 µM) for 4 h. Data are shown as mean(n = 3) ± SEM.

Drug screenings, as exemplified for the known autophagy manipulating drugs Rapamycin, Chloroquine, and Bafilomycin A1, can be readily performed (Fig. 4A–D) using the autophagy reporter cell lines (HeLa-, HEK293T-, Jurkat- and THP-1 eGFP-LC3B). Dotted red lines represent twice the standard deviation of the mock control to illustrate the sensitivity of the approach. Induction of autophagic flux by Rapamycin was visible even at low nanomolar (< 15 nM) concentrations. Accumulation of autophagosomes induced by treatment with Chloroquine or Bafilomycin A1 was detected significantly above background at very low drug concentrations (< 1.25 µM for Chloroquine, < 78.1 nM for Bafilomycin A1), illustrating the sensitivity of the approach. For all treatments, Z-factors ranging between 0.29 and 0.91 were calculated. A Z-factor above 0 indicates high robustness of the high-throughput approach^42^, suggesting that the method is robust enough for high-throughput applications. As a proof-of-principle whether our approach can be used to detect novel autophagy modulating compounds, we assessed the impact of 18 different human amino acids on eGFP-LC3B levels. Our results indicate that whereas amino acids like cysteine, isoleucine, asparagine, serine, valine or threonine may induce autophagy, others like arginine, tyrosine or glycine slightly reduce autophagic flux (Fig. 4E). This is in accordance with previous reports that indicate a role of amino acids in the modulation of autophagy^51–53^. Taken together, all cell lines that were generated responded accurately, robustly, and sensitively to drug treatment. Thus, this approach is suitable for high-throughput quantification of autophagy to discover novel compounds that modulate autophagy (see 3.1.).

### Monitoring modulation of autophagy by viral infections

**Figure 5:**
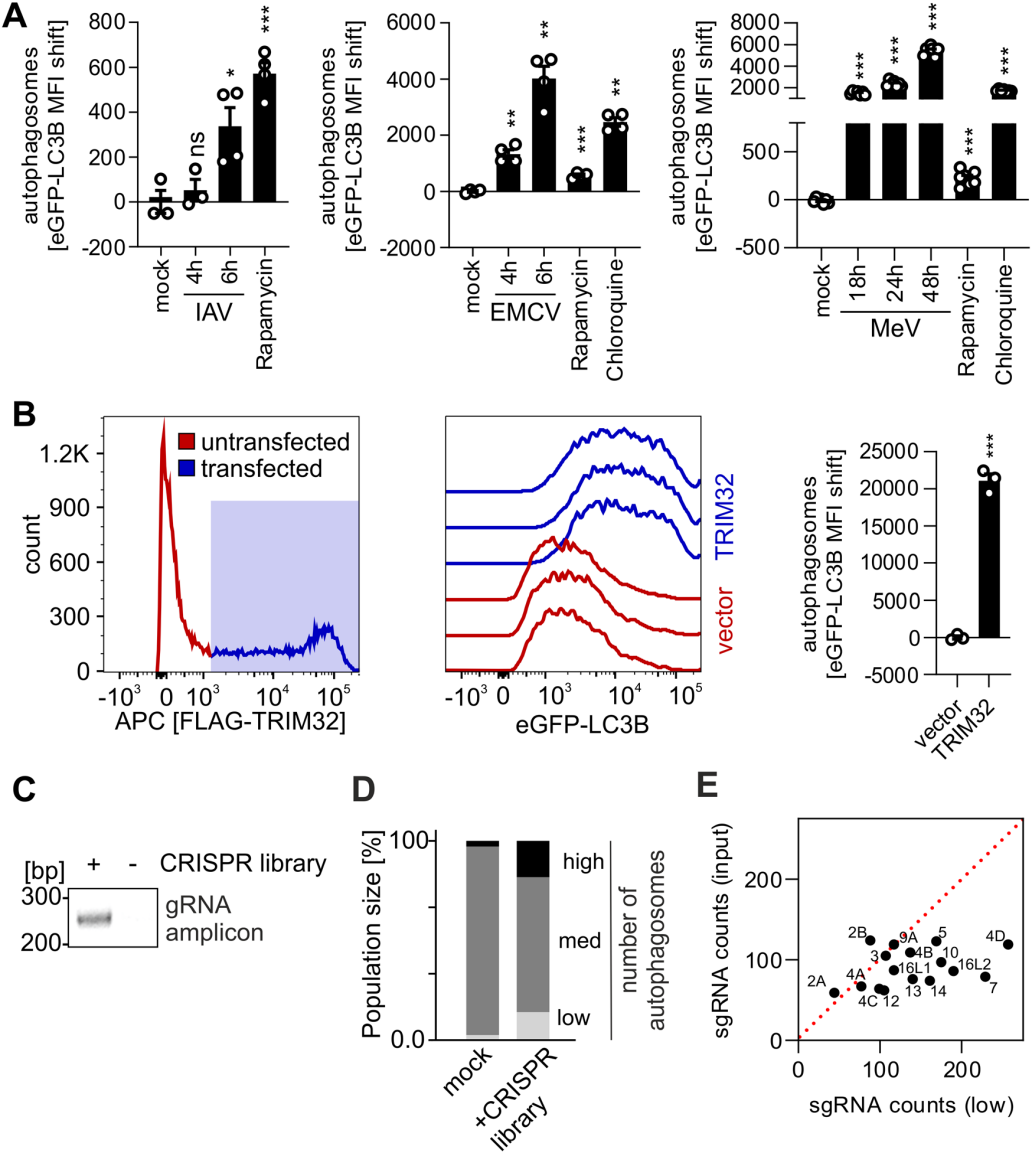
Assessing autophagy during viral infection and overexpression/knockout approaches. (**A**), HeLa GL cells were infected with influenza A virus (IAV, left panel, MOI 5), encephalomyocarditis virus (EMCV, middle panel, MOI 10) or measles virus (MeV, right panel, MOI 5). Cells were harvested, saponin treated, fixed, and the eGFP-LC3B fluorescence analyzed by flow cytometry at the indicated time points post infection. Treatment with Chloroquine (1 µM, 4 h) and Rapamycin (1 µM, 4 h) served as controls. Data are shown as mean(n = 3–6) ± SEM. (**B**), HEK293T GL cells were transiently transfected with an empty vector or a TRIM32-FLAG expressing construct. Cells were saponin treated, fixed, and stained with anti-FLAG antibodies (APC) (left and middle panel). eGFP-LC3B MFI of the transfected cell population was quantified for the TRIM32-FLAG sample and background (= vector) subtracted (right panel). Data are shown as mean(n = 3) ± SEM. (**C**), Agarose gel depicting the sgRNA amplicon amplified by PCR from genomic DNA isolated from saponin treated Jurkat GL cells that were either mock-infected or transduced with the Human CRISPR Knockout Pooled Library (GeCKO v2) (**D**) Exemplary distribution of high/low/medium autophagosome content (= eGFP MFI) in Jurkat eGFP-LC3B cells 10 days after transduction with the Human CRISPR Knockout Pooled Library (GeCKO v2) as assessed by FACS sorting (**E**) Scatter Plot (control population ‘input’ vs low autophagy population ‘low’) of aggregated counts of sgRNAs targeting indicated ATG proteins. Each dot is derived from 6 individual sgRNAs targeting the same gene. The dotted red line indicates an equal abundance of sgRNA counts in both populations. Statistical significance was assessed using unpaired t-test (**A**) or one-way ANOVA (**B**). ns = not significant, *p ≤ 0.05, **p ≤ 0.01, ***p ≤ 0.001. Uncropped agarose gel in Supplementary Fig. 5.

Induction/reduction of autophagosomes upon viral infection was resolved in a time-dependent manner using infected HeLa eGFP-LC3B cells (Fig. 5A). Whereas influenza A virus (IAV) infection significantly increased autophagosome levels in epithelial cells already after 6 h^10,54,55^, measles virus (MeV) infection induced high numbers of autophagosomes at late time points (18 h, 24 h, 48 h)^56,57^. In monocyte-like cells, both IAV and MeV infection induced autophagy at early time points (Fig. S2A). Infection with encephalomyocarditis virus (EMCV) rapidly induced high levels of autophagosomes^10,58^ (Fig. 5A). In summary, time-dependent changes in autophagy induced by viral infection can be accurately monitored on a small scale (96-well) using our system. Furthermore, samples with higher biosafety levels (above BSL1) can be easily processed and fixed, and then safely analyzed in BSL1 conditions (see 3.2.).

### Identification of cellular factors modulating autophagy

Autophagy induction by protein transfection into HEK293T eGFP-LC3B cells can be rapidly assessed as exemplified by inducing autophagy with TRIM32 overexpression^59^ (Fig. 5B in HEK293T reporter cells or Fig. S2B in HeLa reporter cells). Thus, it is possible to screen whole libraries of proteins for autophagy induction (see 3.3.). Similarly, knockdown or knockout libraries using either siRNA or CRISPR/Cas9 can be approached. CRISPR/Cas9 mediated knockout or siRNA mediated knockdown of core components of the autophagic machinery (ATG proteins) led to a clear decrease of the eGFP-LC3B signal (Fig. S2C and D). CRISPR/Cas9 screens require isolation of cell populations that display altered autophagy levels. Subsequently the integrated sgRNA cassettes are amplified and analyzed by next-generation sequencing (NGS) to compare the abundance of sgRNA sequences in the ‘sample’ to the control population (‘input’) and identify target enrichment. We successfully extracted genomic DNA from Jurkat eGFP-LC3B cells after processing and MeOH fixation as described in our basic protocol. Subsequent PCR analysis revealed an amplicon at the correct size (~ 270 nt) that was only present in the sample transduced with a CRISPR Lentivirus library (Human CRISPR Knockout Pooled Library (GeCKO v2)^37,49^, Fig. 5C). As a proof-of-principle for CRISPR/Cas9 screens to identify novel key factors in autophagy in T cells, we transduced Jurkat eGFP-LC3B cells with the Human CRISPR Knockout Pooled Library (GeCKO v2). The samples were processed and fixed with MeOH according to our basic protocol. FACS sorting isolated high, low and medium eGFP-LC3B (= autophagosome) containing cells. 8% of the CRISPR treated cells displayed higher autophagosome content than non-targeting sgRNA infected cells and 10% showed lower levels of eGFP-LC3B (Fig. 5D). Genomic DNA was extracted from ‘low’ and ‘high’ fractions as well as from the control population, ‘input’. The sgRNA cassette was amplified by PCR (Fig. S2E). The amplicons of ‘low’ and ‘input’ from three independent experiments were pooled and sequenced using NGS to identify the contained sgRNAs. From a total 119,461 individual sgRNAs in the original GeCKO library, we could obtain sequences for 97.92% (Fig. S2F), demonstrating that complexity was retained. Knockout of components of the autophagic machinery should lower autophagosome levels. In line with this, sgRNAs targeting ATG genes (6 per gene) were significantly enriched in the ‘low’ population on average (Fig. S2G). Analysis of the aggregated sgRNA counts showed that the counts for large majority of ATG genes were higher in the ‘low’ fraction compared to the ‘input’ (Fig. 5E). This confirms that the method is able to identify components of the autophagic machinery. Taken together, these assays revealed that our method is suitable for high-throughput overexpression or CRISPR/Cas9 mediated KO approaches to discover novel key factors in autophagy (see 3.4.).

## Discussion

### High-throughput quantification of autophagy

A wide variety of methods to reliably quantify autophagy is currently available (for a comprehensive review see^24^). These methods include visualization of autophagosomes by electron microscopy, monitoring of degradation of targets of autophagy such as p62 using western blotting, processing of endogenous LC3B, and visualization of LC3B puncta using eGFP-LC3B and confocal fluorescence microscopy. Advanced imaging methods using automated image processing reduce the manual labor required for confocal image acquisition and analysis^50^. Several currently used methods to quantify autophagy rely on monitoring a hallmark of autophagy induction, the lipidation and translocation of (eGFP-LC3B) to autophagosomal membranes. However, most of these methods are not applicable for high-throughput approaches. Here, we describe a detailed protocol for an easily accessible method to robustly and rapidly quantify autophagosomes for high-throughput applications based on flow cytometry-mediated quantification of membrane (= autophagosome)-bound eGFP-LC3B. Compared to classical methods for monitoring autophagy, like western blotting, this system is less labor-intensive, faster, and allows the quantification of large numbers of cells (10,000 vs 50–100) at once, allowing extraction of robust means of autophagy levels but also visualization of the heterogeneity of cell systems. Therefore, this system is well fitted for approaches that measure the mean induction of autophagy by e.g. drugs or peptides, but also approaches relying on single-cell autophagy levels like CRISPR/Cas9 screens (Figs. 4 and 5). Proof-of-principle assays revealed that novel autophagy modulating compounds like e.g. human amino acids can be readily identified. Alteration in autophagy levels due to viral infections can be assessed over time. Our data further reveal that overexpression approaches monitoring the autophagy response of individual cells are possible. Proteins above a size threshold of approximately 50 kDa can be easily co-stained, allowing e.g. overexpression screenings. To avoid washout of the co-stained protein, it may be anchored via a tag (e.g. GPI anchor) to a membrane and thus be retained in the cell^60^. Lastly, the system is ready for CRISPR/Cas9 KO screens to identify novel factors modulating autophagy in different tissues. We could isolate populations with different autophagy levels in reporter T cells that were transduced with the GeCKO v2 CRISPR/Cas9 pooled library. sgRNA amplicons were extracted and analyzed by NGS. Taken together, we demonstrate that our protocol is suitable for a wide range of high-throughput approaches that can be applied to answer various scientific problems, ranging from pathway analysis and key factor identification to drug discovery.

### Limitations of the method: autophagic flux vs. accumulation of autophagosomes

Autophagy is a highly dynamic process, relying on the complex turnover and activation of signaling cascades. While the reporter cell lines allow fast processing of samples and quantification of autophagy, its use is limited to genetically modified cell lines. The consistent presence of the reporter is mandatory and transiently transfected cell lines do not provide adequate stability of the signal. Thus, the screen should be complemented with monitoring the endogenous LC3B status in primary cells to support conclusions indicated by initial screening methods^24,61^.

One major issue of all systems relying on the processing of LC3B is that both de novo induction of autophagy and blockage of autophagic flux increases the amount of autophagosomes (or processed LC3B) at a given time point. Thus, a rigorous secondary assessment of hits obtained in the primary screen has to take place (for a comprehensive review of methods see^24^). Complementary assays to monitor autophagy, that do not rely on LC3B are mandatory to properly assess the status of autophagy in a cell. For example, the cellular levels of SQSTM1/p62 are decreased upon induction of autophagic flux but p62 accumulates if autophagy is blocked^62,63^. Monitoring p62 levels will provide further hints whether a compound/molecule induces or blocks autophagy^62^. Alternatively, other cellular proteins targeted by autophagy like NBR1^64^ or cGAS^65^ can be used as indicators of autophagic degradation. Autophagy-like cellular processes such as LC3-associated phagocytosis^66^ or viruses may use LC3B and redirect it to membranes, thus giving rise to false-positive signals in our flow cytometry assay and other methods that measure autophagy using LC3B processing or localization^3,54,66^. In general, for the majority of non-canonical autophagic processes, degradation of p62 is not observed. Furthermore, the activity of the compound/factor should be dependent on multiple essential factors of the core machinery of autophagy such as ATG5, ATG4, and ATG16L. For example, while LC3B associated phagocytosis is dependent on most core machinery factors, it is independent of e.g. ATG14L, which is required for canonical autophagy^3,66^. Finally, visual analysis of double-layered membrane structures by electron microscopy will support the notion of whether a compound/regulatory factor modulates classical autophagy^67,68^.

### Comparison to other high-throughput approaches to measure autophagy

Besides relying on eGFP-LC3B, other high-throughput methods to quantify autophagy are available. Cell lines expressing the double fluorescence reporter fusion to LC3B (mRFP-eGFP-LC3B) allow rapid quantification of autophagy without cytoplasmic washout^69^. Upon induction of autophagy, eGFP fluorescence is lost in the acidic environment of autophagolysosomes, thus the ratio between eGFP and mRFP fluorescence decreases. While this method is elegant, it was less sensitive in our hands and required complicated compensation during flow cytometry, which may increase rates of false-positive/false-negative results. Occasionally, eGFP fluorescence is not completely quenched by the acidic pH, resulting in remaining signal, thus alternative fluorophores have been proposed^70^. Still, recently novel autophagy regulating factors like TMEM41B^30^ were discovered using mRFP-eGFP-LC3B reporter constructs^24^.

Recently, another sophisticated approach to quantify autophagic flux using flow cytometry was described^26^. Using a reporter construct expressing a fusion protein of GFP, LC3, RFP, and a non-cleavable variant of LC3B, LC3BΔG, this system allows, similarly to the double-labeled LC3B, monitoring of autophagy based on the ratio between GFP and RFP fluorescence. Upon autophagy induction, the protease ATG4 is activated and cleaves the fusion protein into GFP-LC3and RFP-LC3BΔG. Eventually, only GFP-LC3B can be incorporated into autophagosomes and is degraded. Thus, upon induction of autophagy, the ratio between GFP and RFP fluorescence decreases. This system has allowed the identification of several novel autophagy modulators^26^. However, recombination between the two LC3B ORFs, especially during lentiviral driven applications like CRISPR screens, may render the system inactive.

Instead of assessing the number of autophagosomes to quantify autophagy, the consequences of the induction of autophagic flux can also be monitored by examining p62 levels. Based on this approach several genetic screens have identified novel factors involved in autophagy regulation, such as 6-phosphofructo-2-kinase/fructose-2,6-bisphosphatase^29^, the ufmylation pathway^28^, or the acetyltransferase EP300^27^. However, upon induction of autophagy, transcriptional upregulation of p62 has been observed in some instances. Furthermore, longer assay times are needed to allow the degradation to proceed enough to detect decreased levels of p62.

Lysosomal dyes like lysotracker may be used to stain lysosomes or autophagolysosomes and even to monitor changes in pH^24,71–73^. However, as acidification of lysosomes is not a process exclusive to autophagy induction, such approaches may lead to high false positive/false negative rates. Thus, systems based on lysosomal dyes are rarely used to monitor autophagy.

Taken together, using eGFP-LC3B as a reporter for quantification of autophagy is currently still the most established and advantageous system for quantifying autophagy. It avoids convoluted reporter systems and thus directly quantifies autophagosomes. Our method applies this established tool for high-throughput approaches.

## Concluding remarks

Accurate and robust quantification of autophagosomes by measuring the mean fluorescence intensity of membrane-bound eGFP-LC3B is a powerful high-throughput tool to study autophagy. Our protocol is designed to easily approach e.g. drug discovery, key factor identification, pathway analysis, and virus/host response monitoring. The setup is easy to adopt and provides a robust, flexible, and rapid readout. However, screening results have to be confirmed using orthogonal methods for assessing autophagy levels. We strongly believe our high-throughput approaches may pave the way for the discovery of novel compounds modulating autophagy and provide an immediately accessible and thoroughly tested system for labs to test their compounds/proteins/viruses for autophagy modulation.

## Supplementary information

Supplementary information.
