# Supplementary material for: An improved method for high-throughput quantification of autophagy in mammalian cells
Source: Sci Rep. 2020 Jul 22;10:12241. doi: 10.1038/s41598-020-68607-w (PMC7376206; doi:10.1038/s41598-020-68607-w)
Supplement: Supplementary file 1 — Supplementary information. [file 41598_2020_68607_MOESM1_ESM.pdf]

SUPPLEMENTARY INFORMATION

An Improved Method for High-throughput Quantification of Autophagy in Mammalian Cells

Lennart Koepke, Benjamin Winter, Alexander Grenzner, Kerstin Regensburger, Susanne Engelhart, Johannes A van der Merwe, Stefan Krebs, Helmut Blum, Frank Kirchhoff and Konstantin MJ Sparrer

Supplementary Figure 1 – 5.

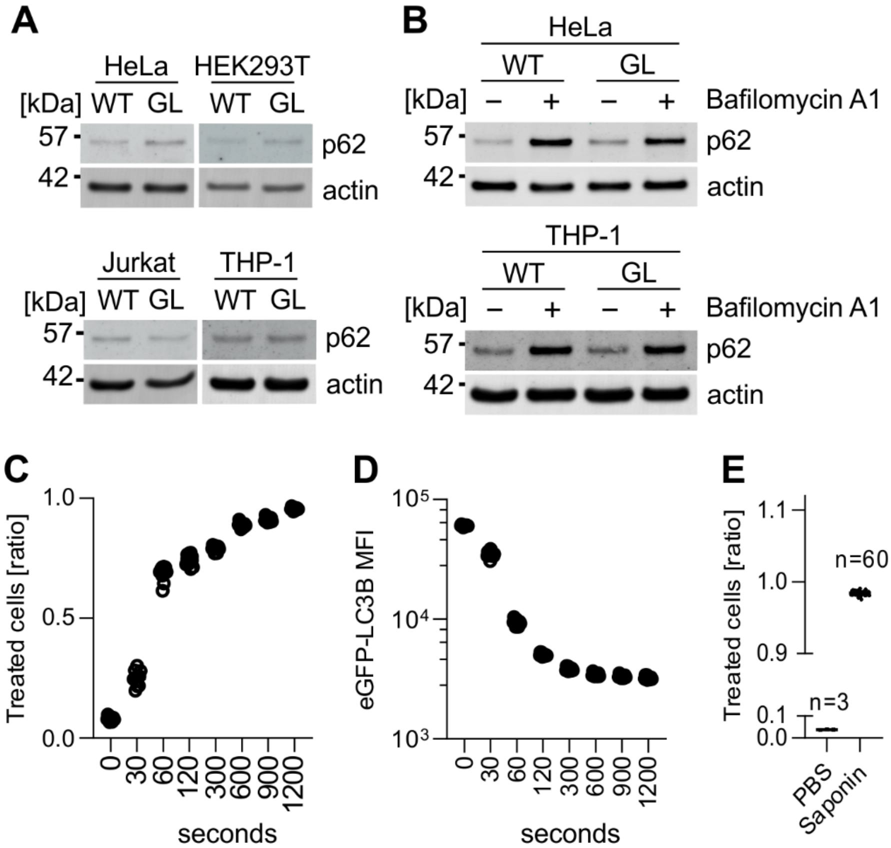

Supplementary Figure 1: Cell line validation. (A), Immunoblot of whole cell lysates of parental (WT) and eGFP-LC3B (GL) expressing cell lines stained with anti-p62 and anti-actin (B), Immunoblot of whole cell lysates of HeLa and THP-1 wildtype (wt) and eGFP-LC3B (GL) expressing cell lines stained with anti-p62 and anti-actin. Cells were treated with Bafilomycin A1 (0.125 µM 18 h) as indicated. (C), Time dependent effect of saponin treatment on the ratio of treated/untreated HeLa cells expressing eGFP-LC3B. Results are shown as mean(n=12)±SEM. (D), Time dependent effect of saponin treatment on eGFP-LC3B MFI in HeLa GL cells after 0, 30, 60, 120, 300, 600, 900 and 1200 s of saponin treatment as analyzed by flow cytometry. Results are shown as mean(n=12)±SEM. (E), Ratio of treated vs. untreated HeLa GL cells after 20 min saponin treatment or 20 min PBS treatment as analyzed by flow cytometry. Results are shown as mean(n=3-60)±SEM. Uncropped western blots in supplementary Fig. 4.

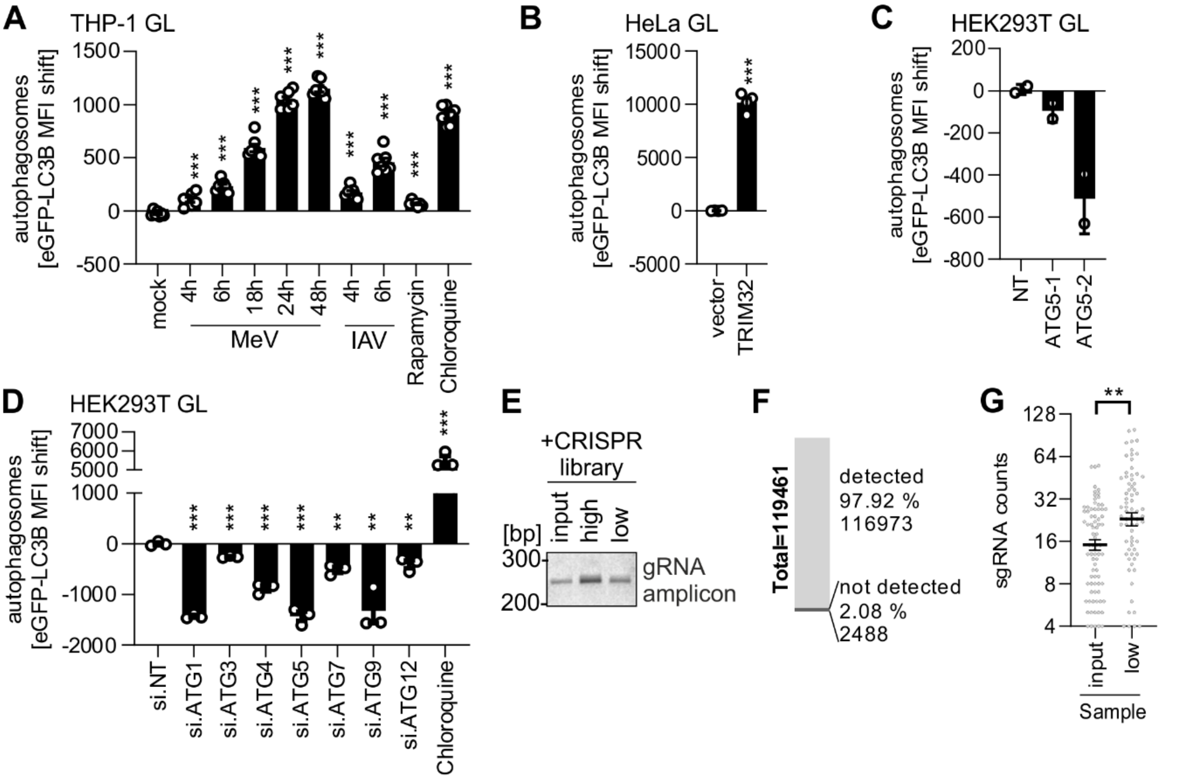

Supplementary Figure 2: Assessing autophagy during viral infection and overexpression/knockdown/knockout approaches. (A) THP-1 eGFP-LC3B cells were infected with influenza A virus (IAV, MOI 5), measles virus (MeV, MOI 5) or left untreated. Cells were harvested, saponin treated, fixed, and analyzed via flow cytometry at the indicated time points. Treatment with Chloroquine (1 µM, 4 h) and Rapamycin (1 µM, 4 h) served as controls. Results are shown as mean(n=7)±SEM. (B), HeLa eGFP-LC3B cells were transiently transfected with an empty vector or a TRIM32-FLAG expressing construct. Cells were saponin treated, fixed, and stained with anti-FLAG antibodies (APC). eGFP-LC3B MFI of the transfected cell population was quantified for the TRIM32-FLAG sample and background (=vector) subtracted. Data are shown as mean(n=3)±SEM. (C), MFI of eGFP-LC3B of saponin-treated HeLa GL cells treated with CRISPR/Cas9 using two sgRNAs targeting *ATG5* (ATG5-1 and ATG5-2). Results are shown as mean(n=2)±SD. (D), MFI of eGFP-LC3B of saponin-treated HeLa GL cells depleted of various ATG proteins using siRNA. Treatment with Chloroquine (10 µM, 4 h) served as control. Results are shown as mean(n=3)±SEM. (E), Agarose gel depicting the sgRNA amplicon amplified by PCR from genomic DNA isolated from either 'input', 'high', or 'low' autophagy population of saponin treated Jurkat GL cells that were transduced with the Human CRISPR Knockout Pooled Library (GeCKO v2). (F), Percentage of detected/not detected sequences of the CRISPR approach in the low autophagy population of the CRISPR/Cas9 screen as assessed by NGS. (G) Distribution of raw sgRNA counts for all individual sgRNA targeting ATG genes (6 per gene). Dots represent one individual sgRNA. The black line indicates the mean(n=96)±SEM. Statistical significance was assessed using unpaired (A,B,D) or paired (G) t-test. ** p≤0.01, *** p≤0.001. Uncropped agarose gels in Supplementary Fig. 5.

HEK293T [WT | GL] & Jurkat [WT | GL] & THP-1 [WT | GL]

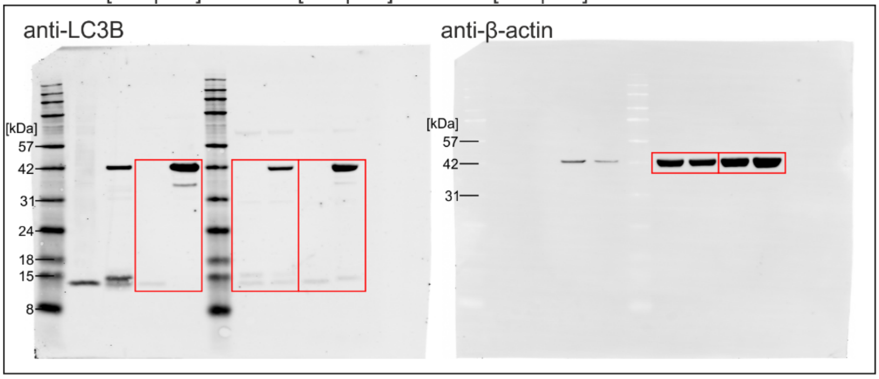

Jurkat [WT | GL] & THP-1 [WT | GL]

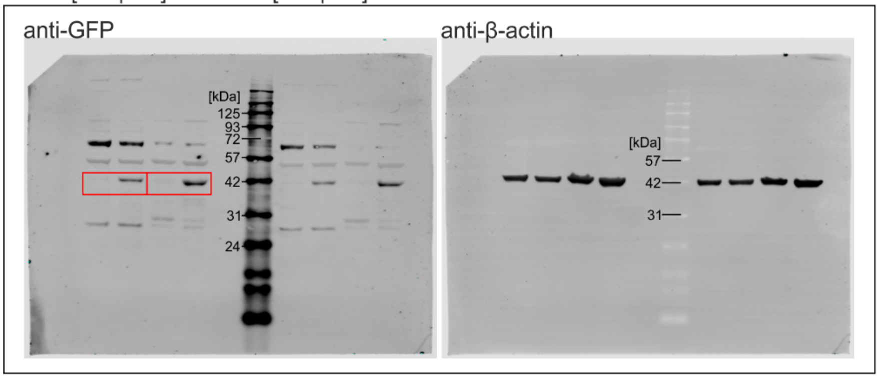

HeLa [WT | GL]

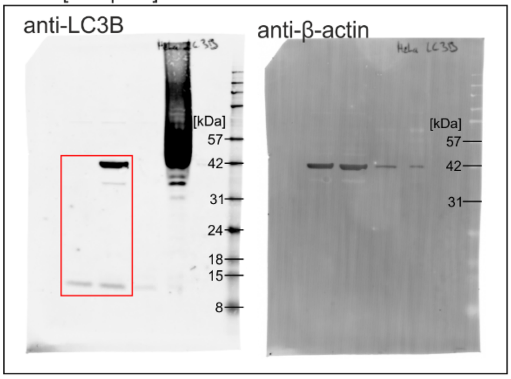

HeLa [WT | GL]

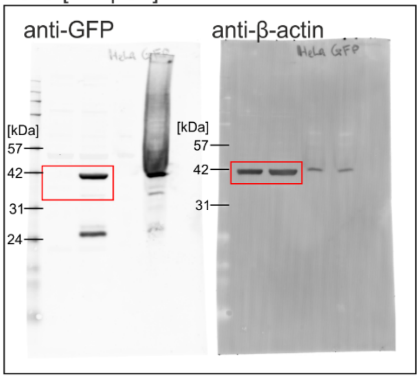

HEK293T [WT | GL]

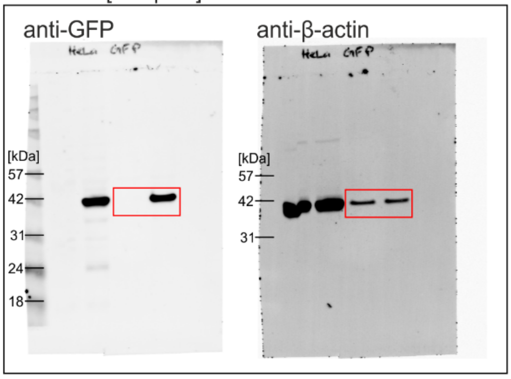

Supplementary Figure 3: Uncropped western blots of figure 1. Black boxes group scans of the same PVDF-membrane. Scans are labelled with the visualized antibody, and areas shown in the figure are marked by red boxes. Size markers are indicated.

HeLa [WT | GL] & HEK293T [WT | GL] & Jurkat [WT | GL]

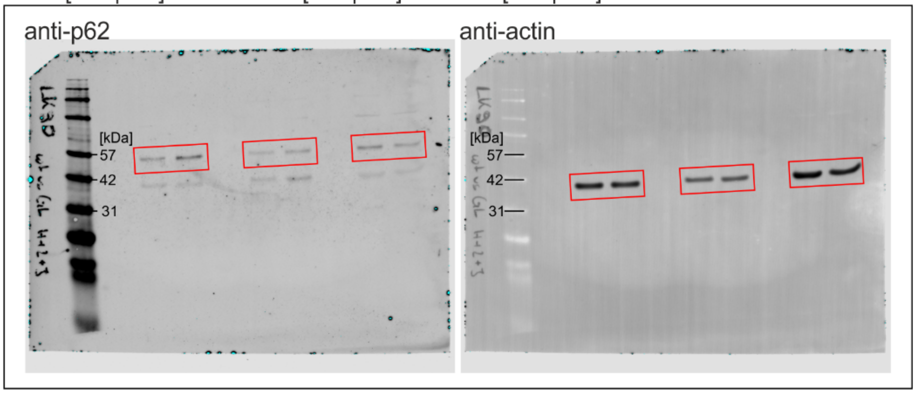

THP-1 [mock | Bafilomycin A1] & THP-1 [WT | GL]

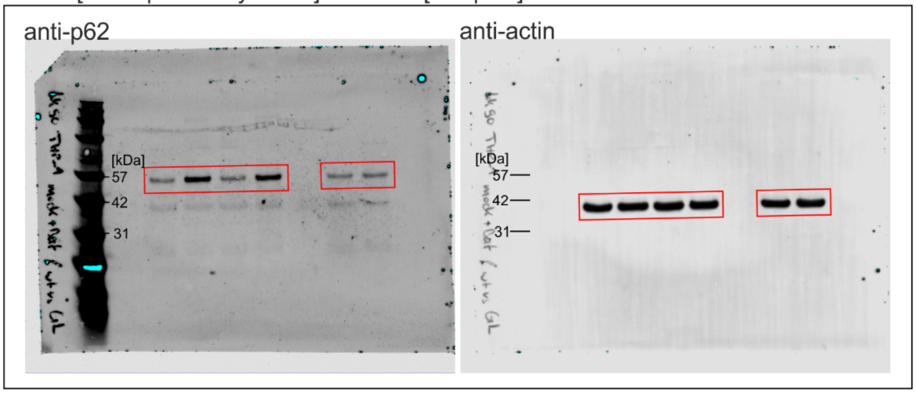

HeLa [mock | Bafilomycin A1]

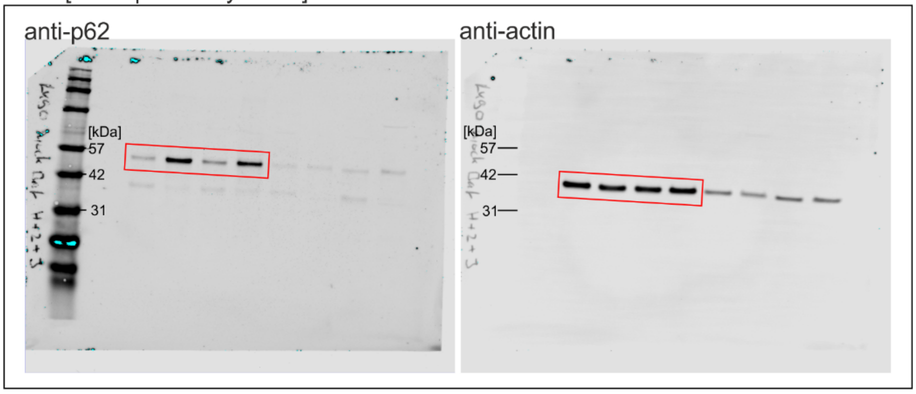

Supplementary Figure 4: Uncropped western blots of supplementary figure 1. Black boxes group scans of the same PVDF-membrane. Scans are labelled with the visualized antibody, and areas shown in the figure are marked by red boxes. Size markers are indicated.

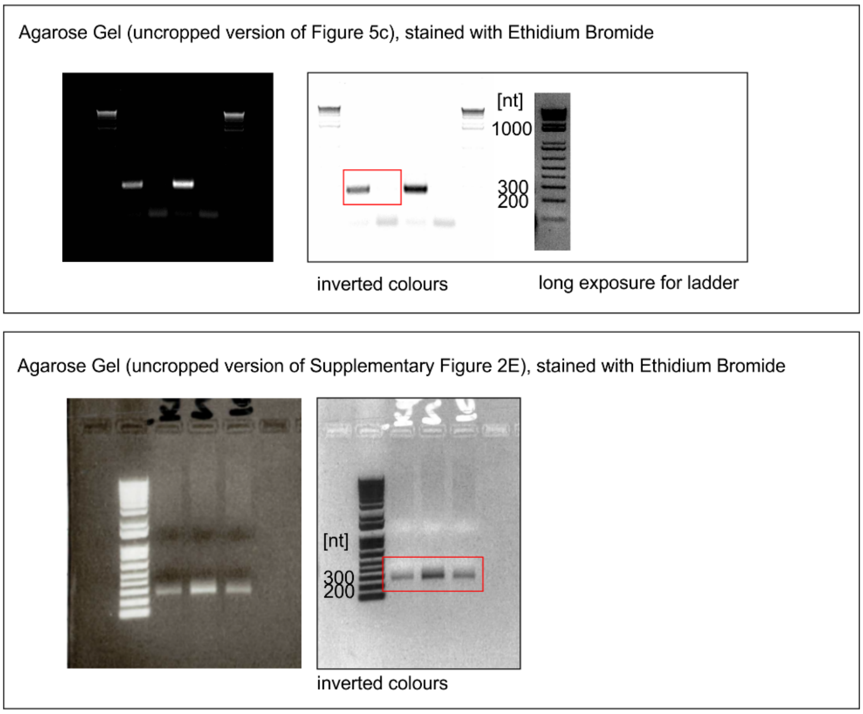

Supplementary Figure 5: Uncropped agarose gels of Figure 5 and Supplementary Figure 2. Black boxes group scans of the same gel. Areas shown in the figure are marked by red boxes. Size markers are indicated.
